# Exploring the xylem-sap to unravel biological features of *Xylella fastidiosa* subspecies *pauca* ST53 in immune, resistant and susceptible crop species through metabolomics and *in vitro* studies

**DOI:** 10.3389/fpls.2023.1343876

**Published:** 2024-01-19

**Authors:** Antony Surano, Carmine del Grosso, Biagia Musio, Stefano Todisco, Annalisa Giampetruzzi, Giuseppe Altamura, Maria Saponari, Vito Gallo, Piero Mastrorilli, Donato Boscia, Pasquale Saldarelli

**Affiliations:** ^1^ Institute for Sustainable Plant Protection, National Research Council (CNR), Bari, Italy; ^2^ Department of Civil, Environmental, Land, Building Engineering and Chemistry (DICATECh), Polytechnic University of Bari, Bari, Italy; ^3^ CRSFA-Centro Ricerca, Sperimentazione e Formazione in Agricoltura Basile Caramia, Locorotondo, Italy; ^4^ Innovative Solutions S.r.l.—Spin-Off Company of Polytechnic University of Bari, Noci, Italy

**Keywords:** olive, xylem-inhabiting pathogen, biofilm formation, bacterial growth, non-targeted NMR spectroscopy, citrus, grapevine, gene expression

## Abstract

*Xylella fastidiosa* subsp. *pauca* ST53 (*Xfp*) is a pathogenic bacterium causing one of the most severe plant diseases currently threatening the olive-growing areas of the Mediterranean, the Olive Quick Decline Syndrome (OQDS). The majority of the olive cultivars upon infections more or less rapidly develop severe desiccation phenomena, while few are resistant (e.g. Leccino and FS17), being less impacted by the infections. The present study contributes to elucidating the basis of the resistance phenomenon by investigating the influence of the composition of the xylem sap of plant species on the rate of bacterial multiplication. Xylem saps from *Xfp* host and non-host species were used for growing the bacterium *in vitro*, monitoring bacterial growth, biofilm formation, and the expression of specific genes. Moreover, species-specific metabolites, such as mannitol, quinic acid, tartaric acid, and choline were identified by non-targeted NMR-based metabolomic analysis in olive, grapevine, and citrus. In general, the xylem saps of immune species, including grapevine and citrus, were richer in amino acids, organic acids, and glucose. The results showed greater bacterial growth in the olive cultivar notoriously susceptible to *Xfp* (Cellina di Nardò), compared to that recorded in the resistant cultivar Leccino. Conversely, higher biofilm formation occurred in Leccino compared to Cellina di Nardò. Using the xylem saps of two *Xfp*-immune species (citrus and grapevine), a divergent bacterial behavior was recorded: low planktonic growth and biofilm production were detected in citrus compared to the grapevine. A parallel evaluation of the expression of 15 genes showed that *Xfp* directs its molecular functions mainly to virulence. Overall, the results gained through this multidisciplinary study contribute to extending the knowledge on the host-pathogen interaction, while confirming that the host response and resistance mechanism have a multifactorial basis, most likely with a cumulative effect on the phenotype.

## 1 Introduction

*Xylella fastidiosa* (*Xf*) Wells et al. (1987) is a gram-negative, aflagellate, strictly aerobic, catalase-positive, oxidase-negative, and non-pigmented bacterium. *X. fastidiosa* and the recently described *X. taiwanensis* (Su et al., 2016) belong to the *Xanthomonadaceae* family, in the class of the *Gammaproteobacteria*, which includes many plant-pathogenic bacteria, mainly of the genera *Xanthomonas* and *Xylella* (Pieretti et al., 2009). A recent survey lists more than 600 plant species in which *Xf* strains have been detected either associated with symptomatic infections and diseases or with latent infections (Delbianco et al., 2023). Diseases of relevant impact on crop species are reported on grapevines (Pierce’s disease), almond trees (leaf scorch), peach (phony peach), citrus (citrus variegated chlorosis), and olives (quick decline syndrome – OQDS) (Janse and Obradovic, 2010; Saponari et al., 2013). Oleander and several shade trees (e.g. *Quercus* spp., *Platanus* spp., *Acer* spp.) upon infection develop typical leaf-scorching symptoms (Janse and Obradovic, 2010). This broad host range results from the high genetic variability characterizing this bacterial species, taxonomically organized in different subspecies (*fastidiosa*, *multiplex* and *pauca* are the most commonly reported) (Nunney et al., 2014) and many strains, which individually can infect a narrower host range. The pathogen, native and well known in the American continent, has recently re-emerged threatening Mediterranean crops and landscapes (Almeida, 2018; Sicard et al., 2018). Outbreaks of the bacterium were reported in Europe for the first time in 2013 when it was discovered in southern Italy in olive trees showing a detrimental disease, the olive quick decline syndrome (OQDS) (Saponari et al., 2013). However, further investigations and genomic studies dated back its establishment in other European countries much earlier than in southern Italy, at least in the second half of the 1900s (Soubeyrand et al., 2018; Landa et al., 2020). This geographical expansion and the colonization of new areas extended the panel of plant species exposed to bacterial infections (Delbianco et al., 2023). Remarkably, the high genome plasticity of the bacterium allowed the successful colonization of novel agricultural and landscape environments (Vanhove et al., 2019).

The advent of the genomic era generated a massive amount of data that contributed to unraveling key features of the genetic traits of *Xf* core and accessory genomes (Castillo et al., 2020). Even with this wealth of genetic information, there are still gaps in knowledge regarding the mechanisms governing host-pathogen interactions (Landa et al., 2022). For instance, it is still unclear which are the factors regulating pathogen lifestyle (i.e. commensalism vs. parasitism) in susceptible host plants, or the complex interactions determining the host specificity for a given strain/genotype combination. Events of recombination are well known to contribute to *Xf* population evolution and adaptation to a new environment (Vanhove et al., 2019), with intersubspecific homologous recombination likely playing an important role in the shifts to novel plant hosts (Vanhove et al., 2019). However, a clear link between *Xf* strain or bacterial sequence type (ST) and host range cannot be predicted based on genomic sequences, and, similarly, it is difficult to identify functional mutations/indels occurring in those genes known to have an important role for the pathogenicity of the bacterium. Despite the long-lasting research programs on this bacterium, one of the most challenging investigations is to understand the mechanisms that are responsible for the incompatibility between a plant species with a given bacterial strain (i.e. immunity). Similarly, the interactions that result in successful host colonization (susceptibility) by a different *Xf* strain need to be better disclosed. An intermediate scenario between immunity and susceptibility is resistance, a phenomenon exploitable for the management of *Xf* diseases, given the lack of therapeutic solutions. Again, there is still a gap in the knowledge about the host genetic traits determining resistance to *Xf.* Several studies have investigated resistance in grapes (Krivanek et al., 2006; Morales-Cruz et al., 2023), citrus (Coletta-Filho et al., 2007; Niza et al., 2015) and, more recently, in olives (Giampetruzzi et al., 2016; Boscia et al., 2017), using different approaches in the attempt to assess the contribution of different factors (anatomical, physiological, and genetic). Regardless of the host species and the bacterial strain, a common feature recorded in these resistant pathosystems is the occurrence of lower bacterial populations as compared to those detected in highly susceptible and symptomatic host plants (Giampetruzzi et al., 2016; Boscia et al., 2017).

This study focuses on *Xf* subsp. *pauca*, ST53 (*Xfp*-ST53), one of the most aggressive bacterial genotypes so far described. An isolate of this genotype was introduced via infected plants, approximately in the early 2000s, in Southern Italy in the region of Apulia, where favorable epidemiological conditions resulted in an epidemic spread of the infections and the associated disease, OQDS, decimating olive trees in the area (Saponari et al., 2017). Unexpectedly, olive turned out to be, among the 36 plant species so far recorded infected by this specific bacterial genotype, the most susceptible. Genomic and microbiological studies have shown several distinctive features of this genotype (Giampetruzzi et al., 2017a; Giampetruzzi et al., 2017b; D’attoma et al., 2020). Despite the severe impact on olive trees, phenomena of resistance to *Xfp*-ST53 have been observed and documented in a few cultivars, namely in Leccino and in FS17 (Boscia et al., 2017; Saponari et al., 2019; Giampetruzzi et al., 2020). Infected trees of these cultivars develop mild symptoms of desiccation and have lower bacterial loads than the highly susceptible cultivars, such as Cellina di Nardò and Ogliarola salentina, or the susceptible cultivar Kalamata (Giampetruzzi et al., 2020), which conversely succumb to the infections.

Several studies explored olive cultivar’s genetics (Giampetruzzi et al., 2016; De Pascali et al., 2019; De Pascali et al., 2022), ionome and biochemical properties (Giampetruzzi et al., 2016; D’Attoma et al., 2019; Sabella et al., 2019), and the xylem vessel anatomy (Walker et al., 2023) to unravel and describe differences between resistant and susceptible cultivars. The main features entailing the resistance responses in a resistant cultivar such as Leccino are the following: (i) it is less susceptible to xylem cavitation than Cellina di Nardò, and more efficient in the refilling mechanisms to rapidly restore the vessel’s hydraulic conductivity (Sabella et al., 2019); (ii) it appears to be more resistant to water stress, eliciting defense pathways against *Xfp* sugars-mediated (De Pascali et al., 2022); (iii) it displays fewer pit membranes degradation compared to the susceptible cultivar Cellina di Nardò upon infections (Montilon et al., 2023); (iv) stomatal conductance and stem water potential are not significantly altered in Leccino, and also in FS17, while resulting heavily affected in Cellina di Nardò (Surano et al., 2022) upon infection; (v) Leccino has a different distribution of vessels diameter than Cellina di Nardò, which makes this resistant cultivar less vulnerable to air embolisms (Walker et al., 2023).

The multiplication of xylem-invading pathogens, such as bacterial, fungal, and oomycete microorganisms, has been reported to be strongly affected by the structure and the nutritional composition of the xylem sap (Yadeta and J. Thomma, 2013). Several studies have explored the *in vitro* growth of *Xf* strains of the subspecies *fastidiosa* (i.e. strains causing Pierce’s Disease) in grapevine xylem sap, in the attempt to mimic in planta conditions and gather information on the host-bacterial interactions, i.e. bacterial phenotype and chemical/molecular mechanisms regulating growth, biofilm formation, aggregation and pathogenesis (Andersen et al., 2007; Cheng et al., 2009; Zaini et al., 2009; Shi et al., 2010; Shi et al., 2013). Despite a relevant number of studies targeting the grape/*Xf* subsp. *fastidiosa* pathosystem, at the best of our knowledge no investigations explored xylem fluids as artificial media to grow *in vitro* strains of the subspecies *multiplex* and *pauca*.

The emergence of OQDS has boosted metabolomic studies to identify host-associated molecules acting as biomarkers of *Xf* infections in olive trees at a very early stage. However, many of these studies have focused on the phytochemical profiles of leaf tissues (Girelli et al., 2019; Asteggiano et al., 2021; Jlilat et al., 2021; Di Masi et al., 2022), while investigations on the metabolic composition of the xylem sap are very limited although it represents the niche colonized by *Xf*. Recent advances in metabolomics have enabled the chemical composition of the xylem sap, in terms of nutrients and secondary/specialized metabolites involved in plant defense against vascular pathogens. Metabolomics was successfully applied to unveil the main metabolites contained in the xylem-sap of two Spanish olive cultivars attempting a correlation of their concentration with the plant age and genotype (Anguita-Maeso et al., 2021). In line with these pioneering works, we attempted to investigate the basis of the resistance mechanism in olives against *Xfp*-ST53, by assessing the *Xfp*-ST53 ability to grow *in vitro* in xylem-sap recovered from susceptible, resistant, and immune plant species. Thus, the bacterial growth and biofilm production of *Xfp*-ST53 were evaluated *in vitro* using variable concentrations of xylem extracted from two different olive cultivars, Cellina di Nardò (*Xfp* susceptible) and Leccino (*Xfp* resistant). In addition, xylem-sap from grape and citrus was also tested as artificial media to grow *Xfp*-ST53, in an attempt to disclose the mechanisms involved in the strain-host plant incompatibility occurring in this specific host-strain combination. *Xfp*-ST53 has never been detected in the epidemic area of Apulia in grapevine and citrus, although infected insect vectors (*Philaenus spumarius*) may visit and feed on plants of both species. These pieces of evidence have been also confirmed experimentally, by artificially inoculating (mechanically or by insect vectors) plants of different cultivars belonging to both species (Potere et al., 2015; Cornara et al., 2017; Saponari et al., 2019). In the present work, growth rates and the ability to form biofilm were compared and studied in relation to the expression of genes involved in the pathogenicity, twitching motility, adhesion, and biofilm formation. Furthermore, the unique capability of the NMR-based non-targeted metabolomic approach of revealing the entire metabolic profile (fingerprint) of a sample has been exploited to identify the biomolecules that influence the growth rate of planktonic cells and the level of biofilm, i.e. the pathogenicity of *Xfp*-ST53. This information can constitute a valid basis for shedding light on the resistance mechanism in olive trees.

## 2 Materials and methods

### 2.1 Plant species and sampling

Healthy trees of three crop species known to be susceptible, resistant, and immune to *Xfp*-ST53, were selected to recover xylem-saps (**Table 1**). Olive, citrus, and grapevine orchards were all located in the same area of the Taranto province (*Xf*-free area of Apulia), characterized by relatively homogenous soil type and climate conditions, and managed according to conventional soil, canopy, irrigation, and fertilization cropping practices. To avoid interference on xylem sap composition, no systemic plant protection products were applied during the growing season. Trees of the olive cultivars Leccino and Cellina di Nardò were grown in the same olive grove.

**Table 1:** Plant species and cultivars selected to recover xylem sap.

Plant species	Response to *Xfp*-ST53	Age of the plants (years)
*Vitis vinifera* cv. Cardinal N.	Immune	**15**
*Citrus sinensis* cv. Navelina	Immune	**25**
*Olea europaea* cv. Leccino	Resistant	**24**
*Olea europaea* cv. Cellina di Nardò	Highly susceptible	**24**

The phenotype response to *Xylella fastidiosa* subsp. *pauca* ST53 (*Xfp*-ST53) is reported.

Sampling was performed in June 2022 when all plants (deciduous and evergreen) were in the full growing season.

Five trees were selected for each cultivar and 5-6 mature hardwood cuttings (diameter ≈ 1 cm) were collected from each tree. Plant materials were collected early in the morning when most of the stomata were still closed, limiting xylem sap losses due to transpiration. Cuttings were transferred and processed within a few hours, stored in darkness with wet paper and at a temperature of 6-8°C.

### 2.2 Xylem sap extraction

Xylem sap extraction from branches was performed with the Scholander pressure chamber Soil Moisture 3000 (Soil Moisture Corp., Goleta, CA, USA) connected to a nitrogen cylinder following the protocol proposed by Alexou and Peuke with some modifications (Alexou and Peuke, 2013). After inserting the branch into the chamber, the bottom side of the main stem was debarked for approximately 2 cm and cleaned with sterile water. The pressure inside the chamber was gradually increased up to a maximum of 35 bars until xylem sap drops were observed on the external portion of the twig. The sap was collected using a Pasteur pipette. From each tree, the yield of xylem-sap varied from 4 to 6 ml. The collected xylem sap was then filtered off through a 0.22 µm sterile filter and the filtrate was kept at -80°C until further analysis. Upon thawing, and before use, the samples were filtered again to ensure microbiological sterility and remove any precipitates and residues resulting from the freezing process. From each tree, an aliquot of 2 ml of xylem-sap was used for NMR analysis, whereas the remaining volume was pooled with the sap of the trees of the same species/cultivar to obtain approx. 15 ml for setting up the *in vitro* growth studies.

### 2.3 Bacterial strain and culture conditions

The strain De Donno of *Xfp*-ST53, originally isolated from an olive showing OQDS symptoms and stored at -80°C in glycerol (50%), was re-cultured and used in our experiments. Bacterial cells were collected from a 7-day-old culture in a PD3 agar plate (Davis, 1992), then resuspended in sterile distilled water and adjusted to an optical density (OD) of 0.5 at 600 nm. A volume of 10 µL of the bacterial suspension was used to inoculate 150 µL of liquid media in single wells of 96-multiwell plates (flat bottom) containing a different ratio of xylem-sap and *X. fastidiosa* medium (XFM) (Killiny and Almeida, 2009). More specifically, all treatments had the same basic nutrient content of XFM at 1x of final concentrations, while the xylem sap varied according to the following final concentrations: 20, 60, and 80%. The medium XFM was selected as it was shown to sustain *Xfp*-ST53 growth at a very low rate (Bodino et al., 2022), thus ensuring that the medium added to the xylem sap had a little, if any, impact on the growth and biofilm formation recorded in the different treatments. Conversely, controlled growth was carried out in PD3 medium, which was used as a reference medium to compare growth and biofilm formation in saps of different plant species. Each treatment included 9 replicated wells and the experiment was repeated in two independent tests. Negative controls corresponded to the same XFM-xylem-sap combinations without the addition of the bacterial suspension. Two different positive controls were prepared by adding the bacterial suspension to 1x XFM and PD3 media without the addition of xylem-sap.

### 2.4 Bacterial growth and biofilm formation

*In vitro* bacterial growth was performed in 96-multiwell plates. After an incubation of 10 days at 28°C with shaking at 165 rpm, the cell growth was estimated by measuring the optical density at 600 nm (OD_600_), using a multi-well plate reader spectrophotometer (TECAN Infinite 200Pro). After taking the OD_600_ measurements, the plates were processed for biofilm determination. The biofilm formation was quantified by using the crystal violet/ethanol protocol (Zaini et al., 2009), with some modifications. Briefly, the plates were rinsed three times with distilled water to remove the planktonic cells. Then, 200 µL of a solution containing crystal violet (0.1% dissolved in water) was added to each well and the plates were kept at room temperature for 20 min. Wells were then washed three times with distilled water before adding 200 µL of absolute ethanol and shaking the plates for 5 min until the crystal violet was completely dissolved. The recovered ethanol–crystal violet solution was measured at OD_600_ nm to estimate the amount of formed biofilm.

Data from all the measured variables were assessed for normality and homogeneity of variance using Bartlett’s test before being subjected to the analysis of variance (ANOVA). When significant, the means of the treatments were compared using Tukey’s test (P ≤ 0.05). Statistical analysis was performed with GraphPad Prism version 9.4.0 for Windows (GraphPad Software, San Diego, California, USA) and RStudio version 1.2.5033 (RStudio, Inc.).

### 2.5 Estimation of the bacterial growth by quantitative PCR

At the end of each experiment, besides measuring the optical density, DNA was extracted from the 10-day-old xylem-sap/media suspensions and subjected to quantitative PCR assay, using the TaqMan-based protocol of Harper et al. (Harper et al., 2010). Briefly, the culture media were collected from the wells, heated at 95°C for 5 minutes, and then rapidly chilled in ice for 5 minutes. An aliquot of 2 µl was directly added in a final volume of 20 µL of qPCR reaction mix (PM 7/24 (5) Xylella fastidiosa, 2023). Estimation of the bacterial population size in these samples was obtained by plotting the recorded quantitation cycles (Cq) on a calibration curve. This curve was generated by preparing three independent 10-fold serial dilutions from an *Xfp*-ST53 suspension OD_600 =_ 0.5 (approx. corresponding to 4×10^8^ CFU/mL), with the dilutions processed as described for the samples.

### 2.6 Gene expression profile of key target genes

A set of *Xf* genes was selected based on previous works (Shi et al., 2010; Beaulieu et al., 2013; Shi et al., 2013) documenting their involvement in different *Xylella* pathways (**Table 2**). Indeed, a new set of primers was designed in the present work based on the genome sequence of *Xylella fastidiosa* “De Donno” (ASM211787v1) (Giampetruzzi, Saponari, Almeida, et al., 2017). Details about the adopted primers are reported in **Supplementary Table S1**. The expression of these selected genes was monitored under the different growing conditions tested in this work.

**Table 2:** Genes of *Xylella fastidiosa* tested for differential gene expression in XFM media amended with xylem sap.

Name	Gene ID	Locus tag (based on Xfp-ST53 “De Donno” genome)	Hypothetical function	References
acvB	PD1902	B9J09_10700	Virulence: protein suggested to regulate pathogenicity and disease symptoms	(Hernandez-Martinez et al., 2006)
algH	PD1276	B9J09_03780	Virulence: exopolysaccharide production, biofilm formation, and maintainance	(Shi et al., 2013)
cvaC	PD0215	B9J09_01180	Virulence: toxin Colicin V encodes a bacteriocin precursor proposed to be a defense mechanism	(De Souza et al., 2003; Pashalidis et al., 2005)
fimA	PD0062	B9J09_00350	Attachment: Fimbrial subunit precursor: component of type I pili important for biofilm formation and cell-cell aggregation	(Feil et al., 2007)
gumB	XF2370	B9J09_03175	EPS synthesis: gumB mutant had reduced ability to form a biofilm in culture but was still able to attach to surfaces, indicating that EPS is involved in biofilm maturation rather than in initial attachment.	(Merfa et al., 2016)
hsf	PD0744	B9J09_07200	Attachment: surface protein adhesion biofilm	(Shi et al., 2010)
hxfA	PD2118	B9J09_11850	Attachment: afimbrial haemagglutinin adhesins, HxfA and HxfB, facilitate cell–cell attachment. Loss-of-function mutations in both the hxfA and hxfB genes result in hypervirulent phenotypes after inoculation into grapevines	(Beaulieu et al., 2013)
hxfB	PD1792	B9J09_10075	Attachment: afimbrial haemagglutinin adhesins, HxfA and HxfB, facilitate cell–cell attachment. Loss of function mutations in both the hxfA and hxfB genes result in hypervirulent phenotypes after inoculation into grapevines	(Beaulieu et al., 2013)
lipB	PD0466	B9J09_08815	Lipoamide biosynthesis: protein modification; protein lipoylation	
pcp	PD1547	B9J09_07130	Membrane component: peptidoglycan associated outer membrane lipoprotein precursor	(Shi et al., 2013)
–	PD1560	B9J09_02325	Virulence: lipopolysaccharide (LPS) biosynthesis	
pglA	PD1485	B9J09_02715	Virulence: polygalacturonase, cell wall degradation enzyme needed for degrading host plant cell walls to allow colonization	(Roper et al., 2007)
pilG	PD0845	B9J09_04895	Twitching: low migration, less motility with respect to XFM (twitching)	(Shi and Lin, 2016)
rpfC	PD0406	B9J09_09125	Virulence: DSF receptor in quorum sensing	(Chatterjee et al., 2008)
xadA3	PD0824	B9J09_04775	Attachment: afimbrial adhesin surface protein	(Feitosa-Junior et al., 2022)

Total RNA was extracted from 3 mL of 10-day-old culture suspensions, using the TRIzol™ Reagent protocol (Invitrogen™). After extraction, total RNA was DNAse-treated using the DNase I protocol (Thermo Scientific™), and cDNA was synthesized using the M-MLV Reverse Transcriptase protocol (Invitrogen™).

Quantitative PCR was set up using a 2x Fast SYBR™ Green Master Mix (Applied Biosystems™) using the standard reaction conditions and 500ng of total RNA template in the reaction mix. The specificity and efficiency of the newly designed primers were preliminarily tested using a 10-fold serial dilution of purified bacterial DNA with a known concentration. The amplification conditions were as follows: 50°C for 2 min, 95°C for 2 min, followed by 40 cycles of 95°C for 15 s, 58°C for 30 s, and 72°C for 30 s. The specificity of each reaction was checked by analysis of the melting curve, consisting of a continuous 0.5°C increase in temperature from 65°C to 95°C. The threshold cycle (Ct) was determined using the default threshold settings. The 2^−ΔΔCt^ method was applied to calculate the relative gene expression levels (Livak and Schmittgen, 2001) using the dnaQ gene as housekeeping (Shi and Lin, 2018). Results are presented as the fold change of target gene expression relative to the reference sample (XFM not amended with xylem sap).

### 2.7 NMR analysis

#### 2.7.1 Chemicals

3-(Trimethylsilyl)-2,2,3,3-tetradeutero-propionic acid, sodium salt (TSP-*d_4_*, CAS N. 24493-21-8, 99%D, Armar Chemicals, Döttingen, Switzerland), hydrochloric acid (HCl, 37%, CAS N. 7647-01-0; ≥99.5%, Sigma-Aldrich, Milan, Italy), deuterium oxide (D_2_O, CAS. N. 7789-20-0, 99.86%D, Eurisotop, Saclay, France) were used for sample preparation. NMR tubes (Norell 509-UP 7) were provided by Norell, Landisville NJ, United States.

#### 2.7.2 Preparation of aqueous extracts for NMR measurements

An aliquot of 600 μL of xylem sap obtained through the procedure reported in paragraph 2.2 was added to 100 µL of TSP-*d_4_*/D_2_O solution [3-(trimethylsilyl)propionic-2,2,3,3-*d_4_* acid sodium salt in D_2_O (0.20%)]. The resulting mixture was vortexed (Advanced Vortex Mixer ZX3, VELP Scientifica Srl, Usmate Velate, Italy) for 1 min at 2500 rpm, and next filtered off through a syringe filter (diameter: 25 mm, pores dimension: 0.2 mm, membrane material: PTFE). The resulting clear solution was poured into an NMR tube.

#### 2.7.3 NMR measurement

1D ^1^H NOESY (Nuclear Overhauser Effect Spectroscopy) spectra were recorded through a Bruker Avance 400 MHz spectrometer equipped with a 5 mm inverse probe. The following acquisition parameters were used to record the spectra. 1D ^1^H NOESY: pulse program = noesygppr1d; size of fid (TD) = 64 K; spectral width (SW) = 20 ppm; transmitter offset = 4.70 ppm; 90° hard pulse (p1) = 8.16 μs; power level for pre-saturation (pl9) = 62.77 dB; dummy scans (ds) = 4; number of scans (ns) = 128; acquisition time = 4.08 s; mixing time (d8) = 0.01 s; recycle delay (d1) = 7 s.

Each spectrum was acquired using TOPSPIN 2.1 software (Bruker BioSpin GmbH, Rheinstetten, Germany) encompassing sample loading, temperature stabilization for 5 min, tuning, matching, and shimming.

NMR raw data (Free Induction Decays, FIDs) were processed using the software MestReNova 11.0 (Mestrelab Research SL, Santiago de Compostela, Spain). The FIDs were zero-filled to 128 K number of points and then underwent the Fourier transformation by applying an exponential multiplication function with a line broadening of 0.1 Hz. Phase and baseline were automatically corrected. The horizontal scale of the chemical shifts is reported in ppm and the TSP-*d_4_* singlet signal, set at δ = 0.00 ppm, was used as a chemical shift reference.

#### 2.7.4 Pre-treatment of raw NMR spectral data for the multivariate statistical analysis

Raw data (FIDs) derived from the 1D ^1^H NOESY measurements were processed by a single operator using Mestrelab and segmented into regular-sized (0.04 ppm) buckets (regular-sized spectral region) in the range of [10.50, 0.50] ppm. The underlying area of each bucket was calculated and normalized to the total intensity, providing the variables to be used in all statistical analyses performed in this study. The areas of the buckets in the region from 5.13 to 4.69 ppm, corresponding to the residual water signal, were set to 0. The data matrix was stored as a table with one sample per row, constituting the observation (a total of 39 samples), and one variable per column (a total of 194 variables). The data matrices were imported into MetaboAnalyst 5.0, and buckets were subjected to mean-centering and divided by the standard deviation of each variable (Unit Variance scaling). Multivariate statistical analyses such as Principal Component Analysis (PCA) and Partial Least Square-Discriminant Analysis (PLS-DA) were performed.

PCA as an unsupervised method was applied to find the directions that best explain the variance in the data set (X) without referring to class labels (Y). The data are summarized into much fewer variables called scores which are weighted averages of the original variables. The weighting profiles, called loadings, were investigated to reveal the most contributing variables to the distribution of the scores. The PCA analysis is performed using the *prcomp* package. The calculation is based on a singular value decomposition. PLS-DA was used as a supervised method that uses multivariate regression techniques to extract, via a linear combination of original variables (X), the information that can predict the class membership (Y). The PLS regression was performed using the *plsr* function provided by *R pls* package (Mevik and Wehrens, 2007). The classification and cross-validation were performed using the corresponding wrapper function offered by the *caret* package (Kuhn, 2020). The caret package is available online: https://topepo.github.io/caret/. To assess the significance of class discrimination, a permutation test was performed. In each permutation, a PLS-DA model was built between the data (X) and the permuted class labels (Y) using the optimal number of components determined by cross-validation for the model based on the original class assignment (Bijlsma et al., 2006). To estimate the predictive ability of the model, the performance measure, Q2, was calculated via cross-validation (CV). In each CV, the predicted data were compared with the original data, and the sum of squared errors was calculated. The prediction error was then summed over all samples (Predicted Residual Sum of Squares or PRESS). The variable importance was measured in PLS-DA through the Variable Importance in Projection (VIP), which is a weighted sum of squares of the PLS loadings taking into account the amount of explained Y-variation in each dimension.

## 3 Results

### 3.1 Evaluation of bacterial growth and biofilm production

**Figure 1:**
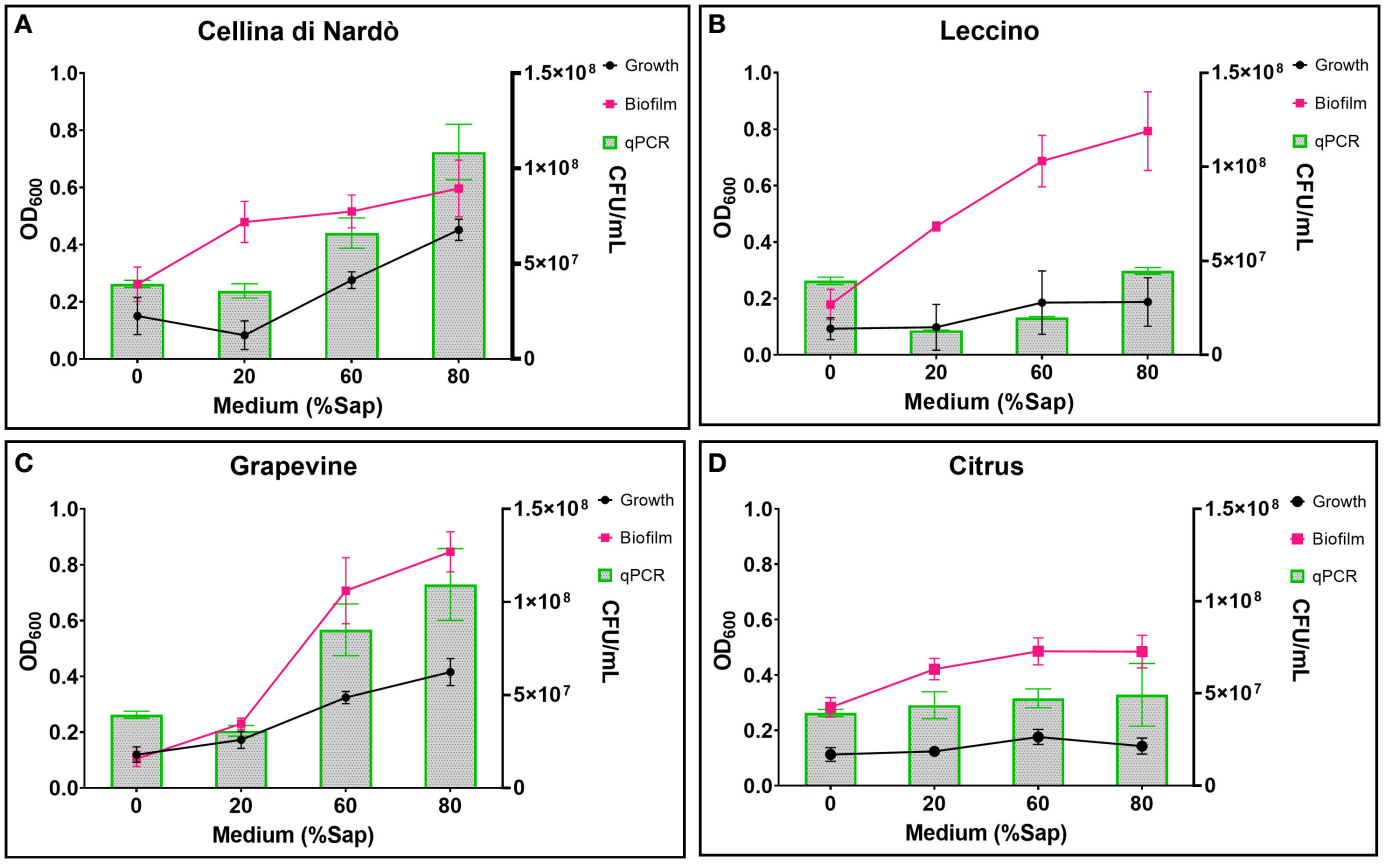
Xfp-ST53 growth and biofilm production in xylem sap of: **(A)** Cellina di Nardò; **(B)** Leccino; **(C)** Grapevine (cv. Cardinal N.); and **(D)** Citrus (cv. Navelina). Growth OD values are multiplied by 10. Xylem sap ratio from 0% (only XFM) to 80%.

Based on the data listed in **Table 3** and **Figure 1**, the addition of increasing amounts of xylem in the range of 20-80% caused, as a general trend, an increase in both bacterial growth and biofilm production. This effect was not found in the case of citrus, for which bacterial growth and biofilm production were found to be almost independent of the sap concentration. On the contrary, a significant increase in bacterial growth was observed for both olive and grapevine samples depending on the concentration of sap, the highest values being recorded when the medium was amended with 80% xylem (3.01-fold for Cellina, 2.02-fold for Leccino, and 3.46-fold for grapevine) (**Table 3**; **Figures 1A–C**). Furthermore, biofilm production was found to be highly dependent on the sap concentration, except in the case of citrus (**Table 3**; **Figure 1D**). Indeed, it was observed that the highest biofilm production in olive and grapevine was achieved when 80% sap was added to the medium. Among the samples investigated, grapevine was the most prone to produce biofilm in the presence of a high amount of xylem, reaching values of 8.11-fold higher compared to those obtained in the absence of xylem (**Table 3**; **Figure 1C**). Also for Leccino the biofilm production was affected by the concentration of sap, reaching values 4.44-fold higher than in the control sample (**Table 3**; **Figure 1B**). For Cellina, the increase in biofilm production reached values of 2.28-fold compared to the control sample (**Table 3**; **Figure 1A**). While for Cellina and grapevine bacterial growth and biofilm production continued to increase at incremental values of sap concentration, for Leccino and citrus almost constant values were observed from 60% of xylem concentration onwards (**Table 3**; **Figure 1**).

**Table 3:** *Xylella fastidiosa* growth and biofilm production recorded as raw OD_600_ values and fold change.

		Medium (% sap)				PD3
		0	20	60	80	
**CELLINA DI NARDO’**	**Growth**	0,0150a	0,0083b	0,0276c	0,0452d	0,0433d
	**Growth fold change**^a^	**1**	**0,55**	**1,84**	**3,01**	
	**Biofilm**	0,2613a	0,4794b	0,5161bc	0,5966c	1.0632d
	**Biofilm production fold change**^b^	**1**	**1,83**	**1,97**	**2,28**	
**LECCINO**	**Growth**	0,0093a	0,0098a	0,0185a	0,0188a	0,0398b
	**Growth fold change**^a^	**1**	**1,05**	**1,99**	**2,02**	
	**Biofilm**	0,1786a	0,4553b	0,6869c	0,7931c	0.9455d
	**Biofilm production fold change**^b^	**1**	**2,55**	**3,85**	**4,44**	
**GRAPEVINE**	**Growth**	0,0120a	0,0173b	0,0323c	0,0415d	0,0518e
	**Growth fold change**^a^	**1**	**1,44**	**2,69**	**3,46**	
	**Biofilm**	0,1044a	0,2304b	0,7070c	0,8465d	1.1531e
	**Biofilm production fold change**^b^	**1**	**2,21**	**6,77**	**8,11**	
**CITRUS**	**Growth**	0,0113a	0,0124a	0,0176b	0,0143a	0.4163c
	**Growth fold change**^a^	**1**	**1,10**	**1,56**	**1,27**	
	**Biofilm**	0,2831a	0,4210b	0,4853c	0,4843c	0.8879d
	**Biofilm production fold change**^b^	**1**	**1,49**	**1,71**	**1,71**	

a Ratio between the bacterial growth observed at different sap concentrations and that of the control at 0% xylem sap (values in bold).

b Ratio between the biofilm production observed at different sap concentrations and that of the control at 0% xylem sap.

**Figure 2:**
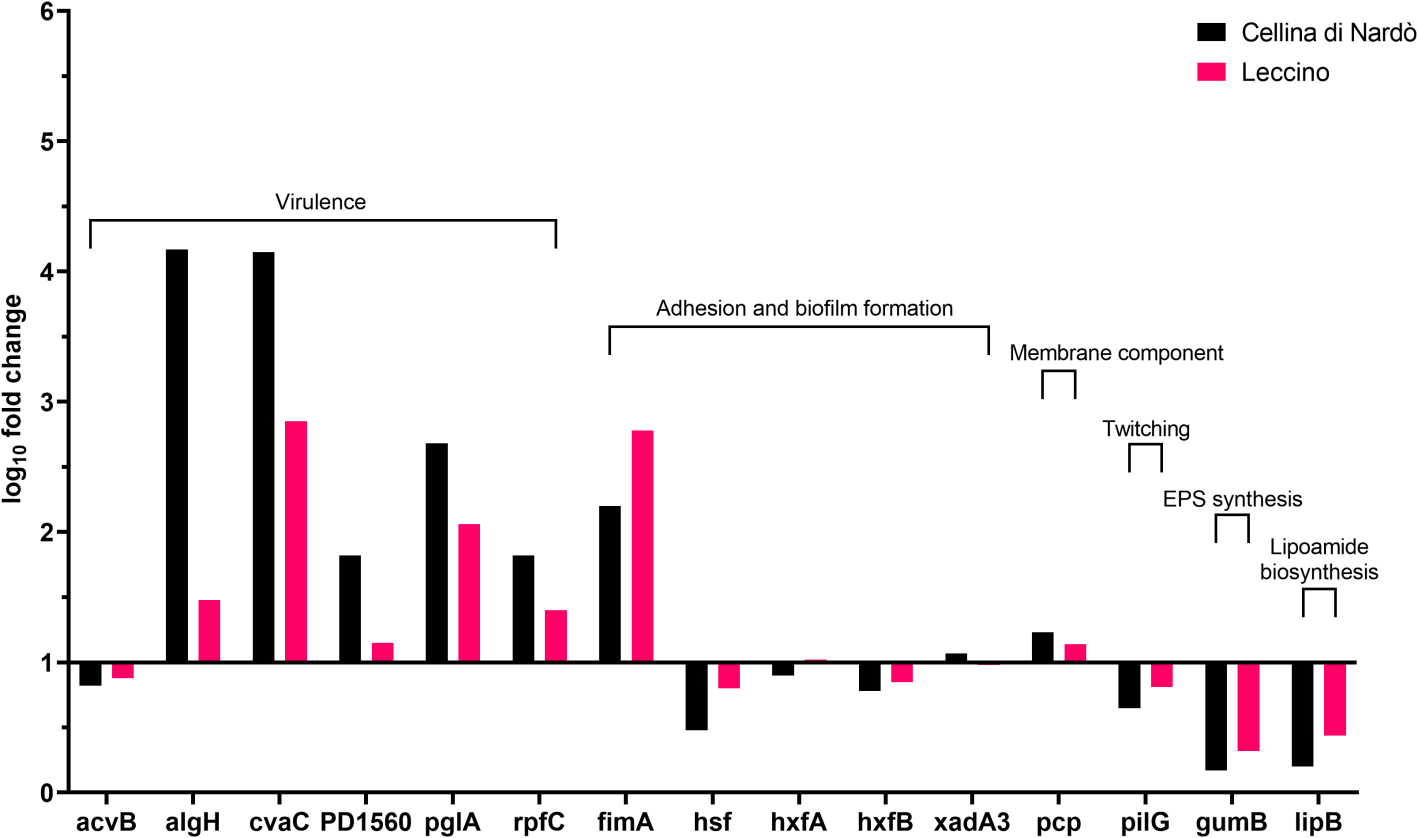
Expression levels of Xylella fastidiosa subsp. pauca “De Donno” genes in XFM medium amended with 80% of xylem sap extracted from Cellina di Nardò and Leccino olive trees.

The highest biofilm:growth ratio was reached in olive when the bacterium was grown in 20% of Cellina di Nardò (79.63) and Leccino xylem saps (81.90) (**Figure 2**; **Table 4**). However, in Cellina di Nardò the biofim:growth ratio decreased with the increase of the xylem sap in the growth medium, with values of 19.08 and 13.34, in 60% and 80% xylem sap concentrations, respectively, with a not significant difference (**Table 4**), likely because of the observed marked increase of growth (**Figure 1A**) in the sap of this olive cultivar. The same decrease of *Xfp* biofilm:growth ratio, although not as with the sap from Cellina, was observed with increasing concentrations of Leccino xylem sap (**Table 4**). In grapevine and citrus, the biofilm:growth ratios at 60% and 80% of xylem sap were respectively more similar to the corresponding values from Cellina di Nardò and Leccino saps (**Table 4**). Indeed, with grapevine sap, the mean values of the ratios were 13.68, 21.95, and 20.59 in 20, 60, and 80% of concentrations, respectively, with no significant differences. In citrus, almost similar ratios (values were 34.73, 28.34, and 35.13 in 20, 60, and 80% of xylem sap concentrations respectively) were observed, with no significant differences.

**Table 4:** Mean values of *Xfp*-ST53 biofilm:growth ratio in different combinations of xylem sap concentrations and source.

	20% xylem sap	60% xylem sap	80% xylem sap
**Cellina di Nardò**	79.63 a	19.08 b	13.34 b
**Leccino**	81.90 a	50.45 ab	51.62 ab
**Grapevine**	13.68 b	21.95 b	20.59 b
**Citrus**	34.73 b	28.34 b	35.13 b

For each combination medium-genotype the data were submitted to two-way ANOVA. Means followed by the same letter are not significantly different according to Tukey’s Test (P ≤0.05).

### 3.2 Gene expression

A set of specific genes was selected based on their role in relevant metabolic pathways of the *Xylella fastidiosa* life cycle. They were classified as associated with virulence, adhesion and biofilm formation, exopolysaccharides synthesis, twitching motility, membrane component, and lipoamide biosynthesis, depending on either the phenotypes of the corresponding available knock-out mutants in *Xylella* or those of orthologue genes from other bacteria species (**Table 2**). Their gene expression was studied in *Xfp*-ST53 grown in 80% of xylem-sap, as this ratio showed the highest differences in growth and biofilm production, in the three plant species and, presumably, better approximated the xylem environment (**Table 3**; **Figure 1**).

Globally, the selected genes follow the same trend of expression in Cellina and Leccino, (**Figure 2**). Five out of six “virulence” genes are upregulated in 80% xylem sap from both cultivars in comparison with their expression in XFM only, which is fixed to a log_10_ fold change value of 1.0 as reference. In particular, algH, a key regulatory gene and cvaC, a putative antimicrobial peptide, are highly induced in Cellina di Nardò in comparison with Leccino, with the first gene having a statistically significant difference in expression (**Table 5**). A moderate but clear upregulation in Cellina di Nardò is also observed for pglA and rpfC, respectively involved in cell-wall degradation and quorum sensing, although without a significant difference (**Table 5**). The unique down-regulated “virulence” gene is acvB. The majority of “adhesion and biofilm formation” genes (hxfA, hxfB, hsf and xadA3) are moderately downregulated or close to their expression in XFM, likely because cells were harvested later in their growth phase when biofilm was mostly already formed. The only exception is fimA, which is highly, although not significantly, induced in Leccino, witnessing the higher biofilm production observed in the 80% amended sap of this cultivar. A further limitedly upregulated gene is pcp, a cell membrane component, perhaps as residual growth of the bacterium while gumB and pilG, having respectively a role in exopolysaccharide (EPS) production and twitching motility are both modestly downregulated, this latter gene, however, significantly differing between Cellina di Nardò and Leccino.

**Table 5:** Expression levels (log_10_ fold change) of *Xylella fastidiosa* subsp. pauca ST53 genes in XFM medium amended with xylem sap extracted from Cellina di Nardò, Leccino, grapevine, and citrus.

	XFM	Cellina di Nardò	Leccino	Grapevine	Citrus
acvB	1.000 a	0.819 ab	0.879 ab	0.656 b	0.883 ab
algH	1.000 a	4.174 b	1.484 a	5.009 b	1.259 a
cvaC	1.000 a	4.153 bc	2.852 abc	5.153 b	1.809 ac
fimA	1.000 a	2.200 b	2.778 b	2.317 b	2.883 b
gumB	1.000 a	0.174 bc	0.322 bd	0.098 c	0.326 d
hsf	1.000 a	0.477 bc	0.798 ab	0.472 c	0.562 bc
hxfA	1.000 ab	0.897 a	0.990 ab	0.528 c	1.115 b
hxfB	1.000 ns	0.775 ns	0.849 ns	0.689 ns	0.896 ns
lipB	1.000 a	0.200 b	0.439 c	0.155 b	0.354 c
pcp	1.000 ns	1.229 ns	1.135 ns	1.085 ns	0.763 ns
PD1560	1.000 a	1.816 b	1.148 ac	1.574 bc	1.211 ac
pglA	1.000 a	2.682 b	2.056 bc	2.934 b	1.488 ac
pilG	1.000 a	0.651 b	0.808 c	0.541 b	0.840 c
rpfC	1.000 a	1.823 b	1.399 ab	1.861 b	0.974 a
xadA3	1.000 ns	1.073 ns	0.976 ns	1.166 ns	1.084 ns

Values represent the mean fold change gene expression of each target gene relative to the housekeeping gene (dnaQ). Means per row were submitted to one-way ANOVA (P ≤ 0.05) if significant values followed by the same letter in the row are not significantly different according to Tukey’s Test (P ≤ 0.05).

**Figure 3:**
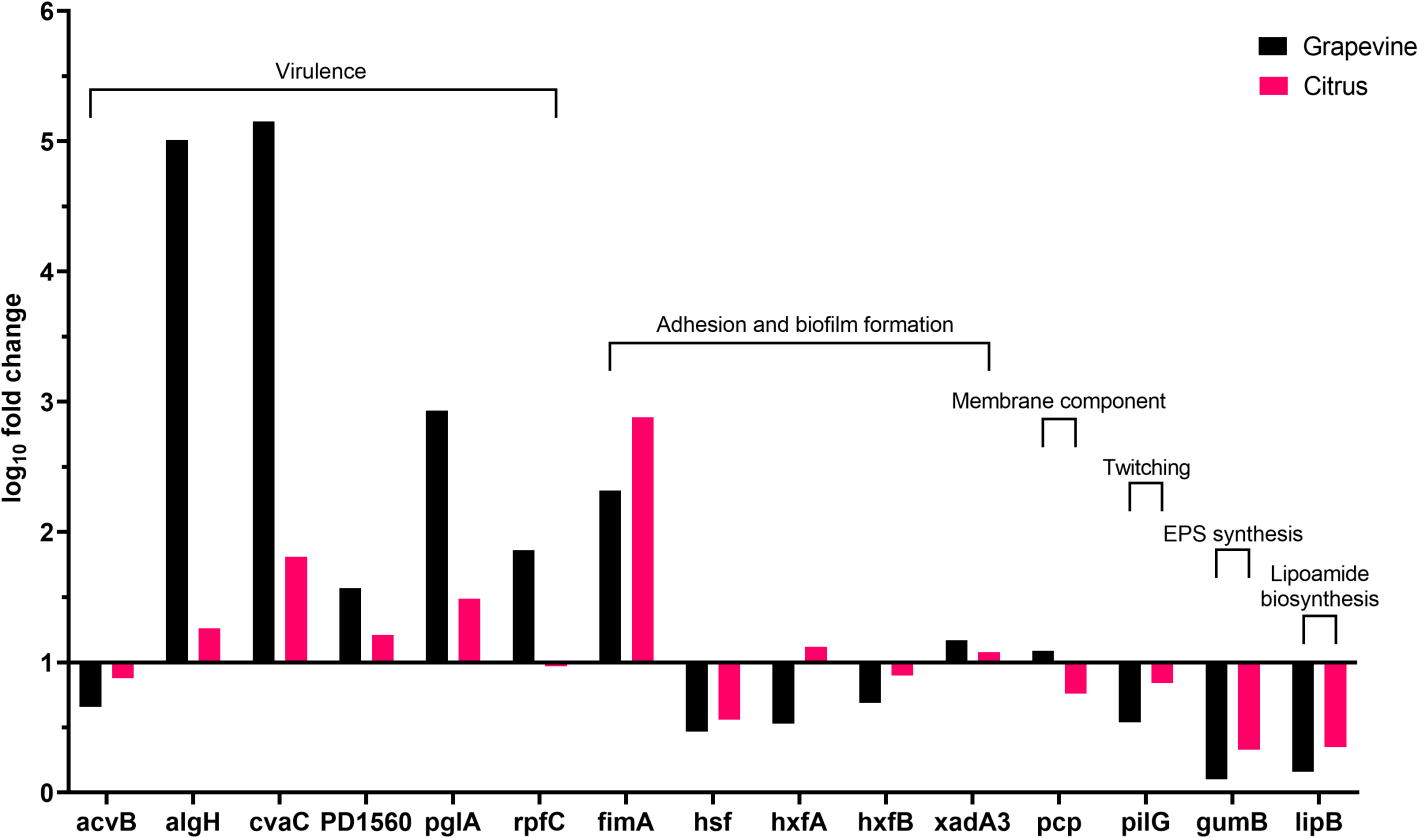
Expression levels of *Xylella fastidiosa* subsp. *pauca* genes in XFM medium amended with 80% of xylem sap extracted from grapevine cv. Cardinal N. and *Citrus sinensis* cv. Navelina.

Besides following the same global tendency of gene expression observed in olives, higher values are obtained from the bacterium grown in grapevine sap in comparison with values of the bacterium cultivated in citrus sap (**Figure 3**). Indeed, the high population size and biofilm amount (**Figure 1C**) are reflected by the values of gene expression observed in grapevine sap (**Figure 3**). Conversely, the low values of both bacterial growth and biofilm formation observed in citrus sap (**Figure 1D**) are mirrored by the low gene expression values (**Figure 3**) which did not largely differ from those obtained in XFM only. However, a significantly higher gene expression of “virulence” gene algH, cvaC, pglA, and rpfC is observed in grapevine compared to citrus (**Figure 3**; **Table 5**). While genes involved in adhesion and biofilm formation (hxfA and fimA) are significantly upregulated in bacteria cultivated in citrus sap (**Figure 3**; **Table 5**). Conversely, genes involved in exopolysaccharides synthesis (gumB), adhesion and biofilm formation (hsf), twitching motility (pilG), and lipoamide biosynthesis (lipB) are downregulated in *Xfp*-ST53 cultivated in citrus sap.

### 3.3 NMR analysis of the xylem saps obtained from olive, grapevine, and citrus trees

**Figure 4:**
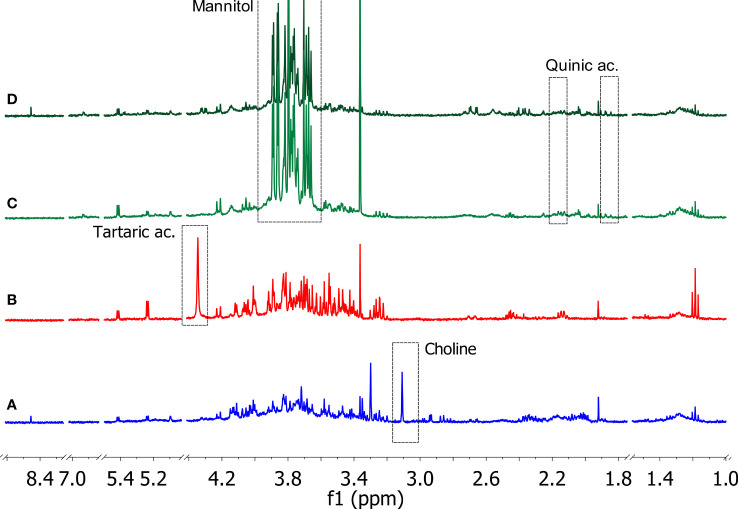
Typical 1D ^1^H NOESY spectra of xylem saps from Citrus sinensis cv. *Navelina (**A**, blue)*, Vitis vinifera cv. *Cardinal N. (**B**, red)*, Olea europaea cv. *Leccino (**C**, light green), and* Olea europaea cv. *Cellina di Nardò (**D**, dark green).* The residual water signal (4.78 ppm) is hidden (Bruker Avance 400 MHz, D2O). Characteristic signals related to species-specific metabolites are indicated in the dashed rectangles.

The 1D ^1^H NOESY measurements provided useful information on the changes in the metabolic composition of the xylem sap derived from the three crop species, i.e. olive, grapevine, and citrus. Typical 1D ^1^H NOESY spectra related to each type of sample are reported in **Figure 4**. All the samples were characterized by significant levels of metabolites belonging to the following classes of compounds: alcohols, carbohydrates, organic acids, and amino acids. From a thorough inspection of all produced spectra, some species-specific metabolites such as tartaric acid, choline, quinic acid, and mannitol were detected in the xylem saps. In particular, tartaric acid was found only in xylematic samples derived from *Vitis vinifera*, choline was significantly detected only in the xylem sap from *Citrus sinensis* trees, while quinic acid and mannitol characterized specifically the xylematic samples extracted from *Olea europaea* trees.

The other identified metabolites were contained with variable amounts depending on the plant species of origin and, in the case of the olive samples, on the cultivar of origin, i.e. Leccino or Cellina di Nardò (see **Supplementary Figures S1–S4** and **Supplementary Tables S2–S5** for further details). An in-depth study of the obtained spectra was devoted to revealing the variations of the metabolic composition in the samples under investigation. Ethanol and methanol were the most representative alcohols, where the former was found to be more abundant in the xylem sap of the grapevine, while the levels of the latter were higher in the xylem sap extracted from Leccino. Among the organic acids, lactic, citric, malic, acetic, formic, quinic, pyruvic, and tartaric acids were identified. The xylem sap extracted from citrus trees contained most of these organic acids, except for quinic (only traces) and tartaric acids, which were specifically found in the xylem saps extracted from the olive tree and the grapevine, respectively. Many organic acids (citric, malic, acetic, formic, quinic) were also detected in the xylem sap extracted from Cellina di Nardò. Conversely, the sap extracted from Leccino showed a low content of these metabolites, except for malic, quinic, and pyruvic acids. Carbohydrates such as glucose, fructose, and sucrose were found uniformly in the samples under study. Mannitol was instead contained exclusively in the xylem sap extracted from olive trees. It was found that, among the amino acids, alanine and threonine were uniformly represented in the different samples, unlike the other amino acids whose content was variable within the various samples analyzed. Specifically, valine was detected exclusively in samples extracted from grapevine. Furthermore, proline was not detected in the xylem sap extracted from olive trees, regardless of the cultivar of origin.

### 3.4 Statistical analysis of the 1D ^1^H NOESY spectral data of xylem saps

**Figure 5:**
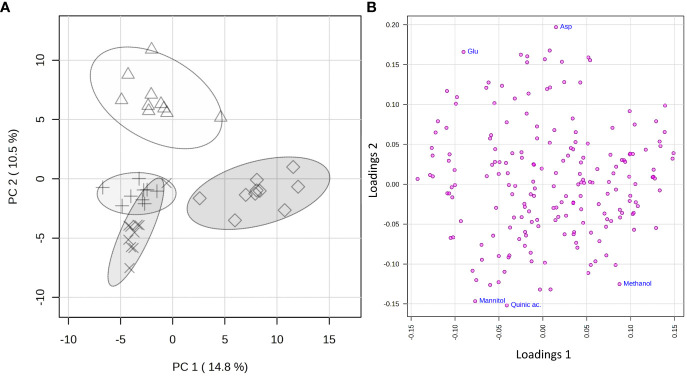
PCA applied to the 325 spectra by using UV-scaled 0.04 ppm-sized bucketing. **(A)** Scores plot between the selected PCs where the observations are indicated according to the belonging species: Δ for *Citrus sinensis* cv. Navelina, + for *Olea europaea* cv. Cellina di Nardò, × for *Olea europaea* cv. Leccino, and ◊ for *Vitis vinifera* cv. Cardinal N., respectively. The explained variances by individual PC are shown in brackets. **(B)** Loadings plot for the selected PCs.

The statistical analysis was applied to a data matrix consisting of 39 rows (samples) and 194 columns (variables). Upon normalization to constant sum and auto-scaling, the obtained data were subjected to the PCA to obtain a preliminary overview of features that are potentially significant in discriminating the samples. The first two principal components (PC1 vs. PC2) explained 25.3% of the accumulated variance with a noticeable clustering of the observations in the scores plot (**Figure 5A**). A clear grouping of the samples according to the species to which they belong was observed.

To reveal the metabolic biomarkers associated with the different phenotypes recorded for the different plant species, a more in-depth analysis of the metabolic composition of the xylem extracted was performed. Based on the study of the loading plot (**Figure 5B**) and the 1D ^1^H NOESY spectra of the samples, methanol was found to be associated with the grapevine, while mannitol and quinic acid to olive, regardless of the cultivar. On the other side, the xylem sap extracted from citrus was more abundant in aspartic and glutamic acids.

**Figure 6:**
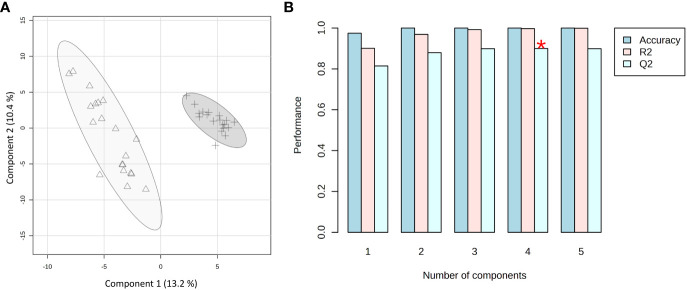
PLS-DA applied to the 39 spectra by using UV-scaled 0.04 ppm-sized bucketing. **(A)** Scores plot between the selected components where the observations are indicated according to the plant behavior towards the *Xylella fastidiosa* infection: Δ for immune, and + for not immune (including resistant and susceptible), respectively. The explained variances are shown in brackets. **(B)** The 10-fold cross-validation method was applied to the PLS-DA model to select the optimal number of components for classification and estimate the predictive performance of the classification model. The red star indicates the component with the highest value of accumulative Q2.

Based on the phenotype response to *Xylella* infection, the samples were grouped into two classes: immune (*C. sinensis* and *V. vinifera)* and not immune (the two olive cultivars, as both support *Xfp*-ST53 multiplication). PLS-DA was applied as a supervised method to extract the information capable of predicting the belonging class of the samples (immune *vs.* not immune) through a linear combination of original variables (spectral buckets). The model presented five components. The developed model was characterized by good predictions and without overfitting as demonstrated by a value of Q2 higher than 0.82 (**Figure 6B**). The first two components exerted the highest contribution with an explained variance of 23.6%. Indeed, the PLS-DA scores plot between Component 1 and Component 2 clearly shows two clusters along Component 1 with an explained variance of 13.2% (**Figure 6A**).

**Figure 7:**
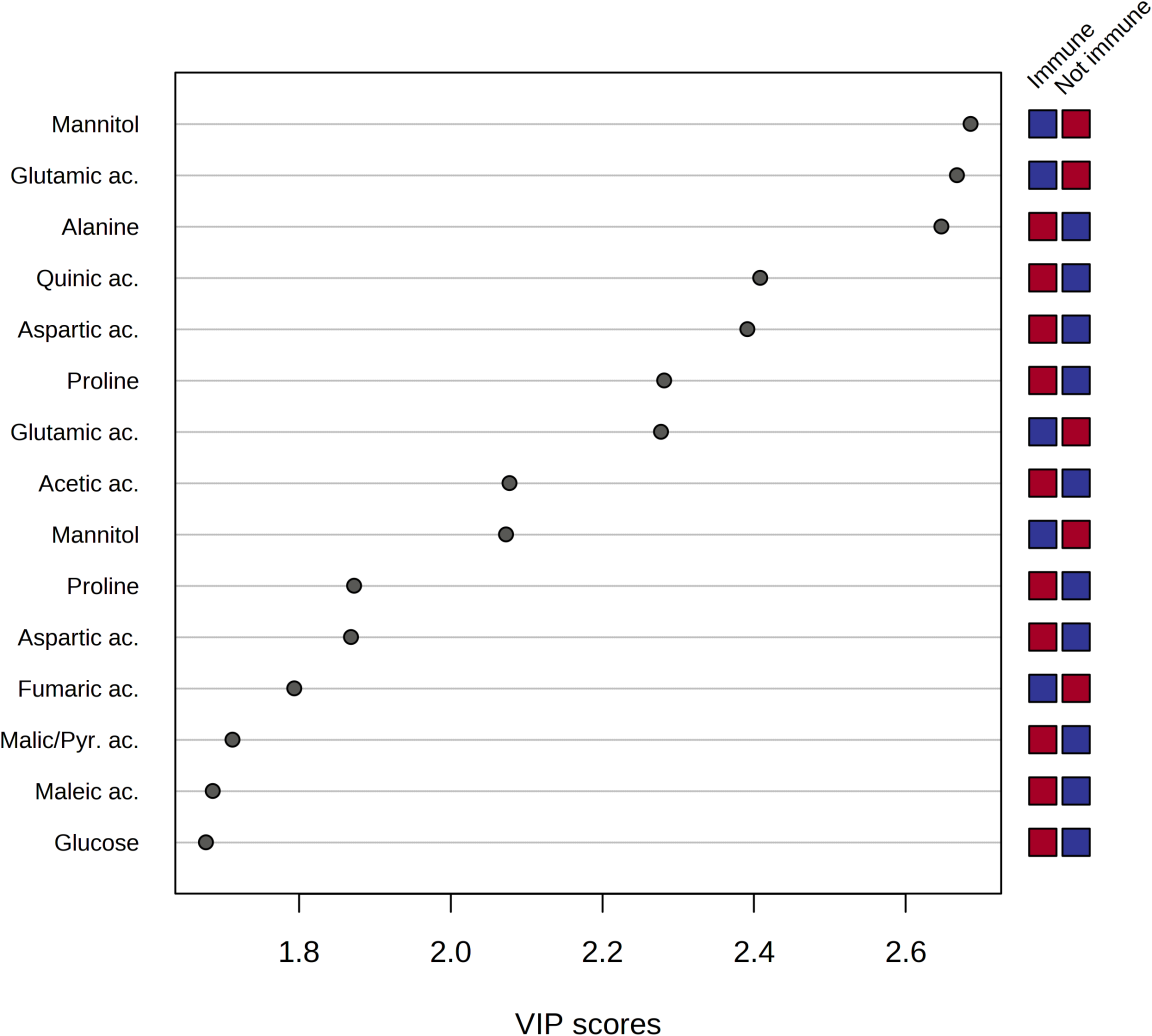
Variable importance analysis in the projection (VIP) identified by PLS-DA where samples are indicated based on the behavior of the plant towards *Xylella fastidiosa* infection, i.e. immune and non-immune (including resistant and sensitive), respectively. The colored boxes on the right indicate the relative mean signal intensity of the corresponding metabolite in each study group (immune vs. not immune), i.e. red and blue for relatively higher and lower intensities, respectively.

The analysis of the Variable Importance in Projection (VIP) was performed to get insights into the variables, and, thus, the metabolites which mostly contributed to the observed distribution of xylem samples in the PLS-DA scores plot. As a result, a pool of 50 variables with VIP > 1 on Component 1 was identified. Among them, fifteen spectral regions were selected as the most discriminating between the two groups of samples, and, thus, fifteen metabolites were identified as potential biomarkers characteristic of sap derived from the immune plants and not immune ones, respectively (**Figure 7**).

The analysis of the VIP plot indicated that the xylem sap extracted from not immune species contained a significant amount of mannitol, ethanol, quinic, fumaric, and lactic acids. On the other hand, the metabolic composition of the xylem sap from immune species resulted in a richer pool of metabolites, including amino acids such as glutamic acid, alanine, aspartic acid, proline, and threonine. Besides, the organic acids, such as acetic, maleic, formic, and pyruvic acids, were contained in a higher amount in the xylem sap obtained from the immune plants.

**Figure 8:**
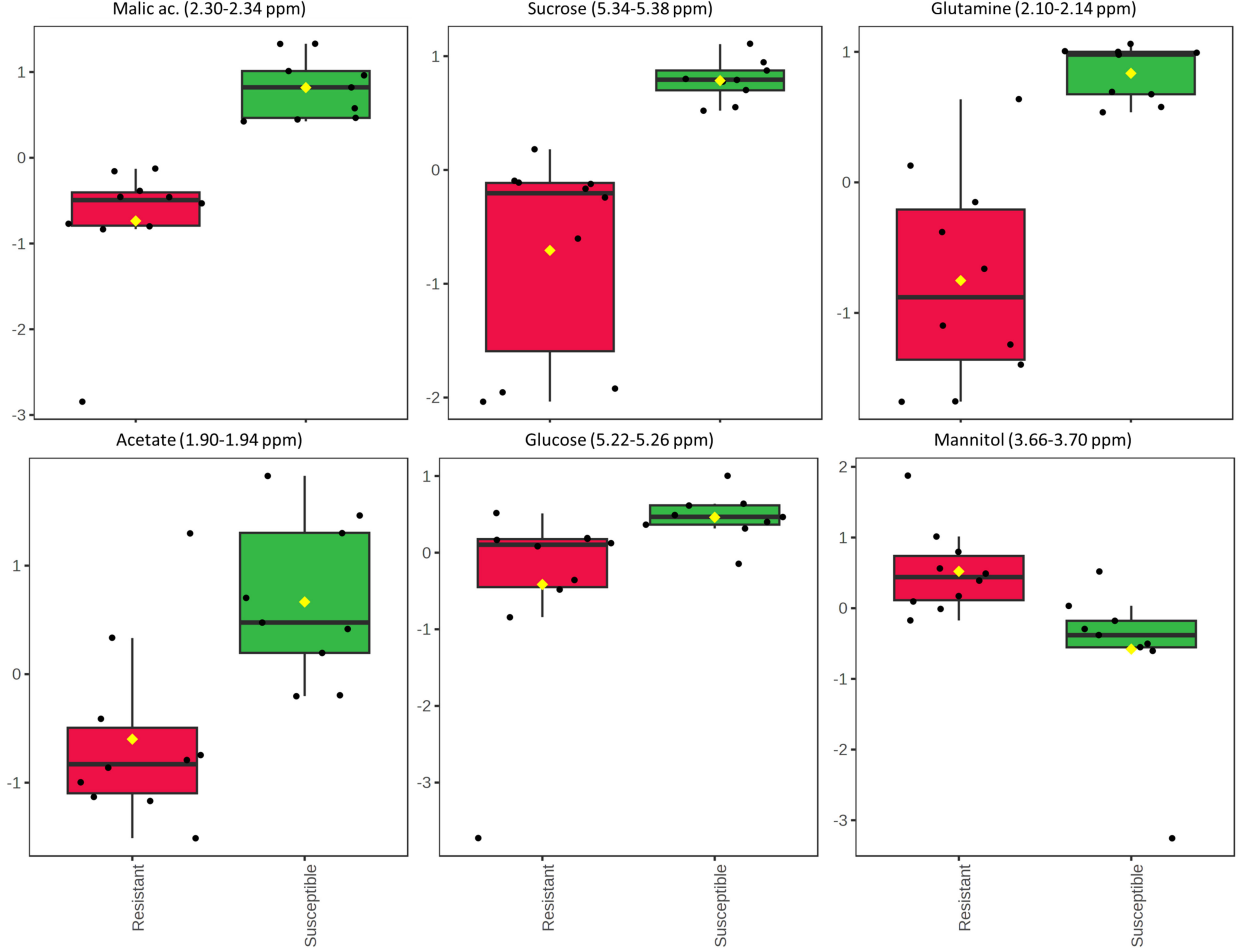
Results of the Wilcoxon rank test performed on the xylem sap extracted from the two olive cultivars Leccino and Cellina di Nardò. The relative intensities of the first six most significant normalized variables is shown according to the Wilcoxon rank sum test imposing a threshold of *p* = 0.05. Each boxplot refers to a bucket in the NMR spectrum (variable), containing the signals assigned to a specific metabolite. In each boxplot, the dots represent the samples and the boxes represent the different phenotype considered for the two olive cultivars, i.e. resistant and susceptible. The boxes are colored red and green for resistant and susceptible phenotypes, respectively.

Next, the statistical analysis was focused on the two olive cultivars Leccino and Cellina di Nardò, taking into account that the first shows a resistant phenotype and the latter a susceptible phenotype. The unpaired two-sample Wilcoxon test (also known as the Wilcoxon rank-sum test or Mann-Whitney test), as a non-parametric alternative to the unpaired two-sample *t*-test, was used to compare the samples recovered from these two olive cultivars. As a result, 30 significant features with a *p*-value threshold of 0.05 were detected. Among these features, the six most significant spectral regions were assigned to the following metabolites: malic acid, sucrose, glutamine, acetate, glucose, and mannitol. All of them, except for mannitol, were found in a higher concentration in the xylem sap of Cellina di Nardò (**Figure 8**).

## 4 Discussion

In this work, we successfully explored and optimized the use of xylem fluids recovered from different plant species as artificial media to study the *in vitro* behavior of *Xfp*-ST53 strain De Donno. In particular, xylem-saps of susceptible (Cellina di Nardò) and resistant (Leccino) olive cultivars were tested along with species known to be immune to *Xfp*-ST53 infections (citrus and grapevine).

We found that the xylem saps of Cellina di Nardò and Leccino differently affected the planktonic growth of the strain De Donno. Under our experimental conditions, the bacterial growth positively correlated with the % of the xylem of sap Cellina di Nardò (y = 0.0192x - 0.0121; R² = 0.997). Similarly, in the same treatment, the production of biofilm increased when the content of xylem sap increased from 20% to 80%, (y = 0.059x + 0.4127; R² = 0.9557). In Leccino, regardless of the xylem sap concentration, the total bacterial growth was lower than in Cellina di Nardò. Despite this low and constant bacterial growth, the xylem sap of this resistant cultivar promoted biofilm formation as its concentration increased (y = 0.169x + 0.307; R² = 0.9557). This suggests that Leccino and Cellina di Nardò xylem saps lead to a different growth behavior, with a prevalence of planktonic cells in Cellina di Nardò compared to Leccino.

In the case of citrus, regardless of the xylem sap concentration, bacterial growth is comparable to that of the XFM minimal medium, used as a control. In fact, the addition of citrus sap determines an almost null effect on growth and a modest effect on biofilm formation which, however, reaches a plateau at the highest concentrations of sap. This behavior may be ascribed to the citrus immunity to Xfp-ST53. Even if grapevine and citrus are both immune to *Xfp*-ST53, we found consistent differences in bacterial growth in the presence of incremental amounts of xylem-saps recovered from these two species. In the case of citrus, regardless of the xylem sap concentration, bacterial growth is comparable to that of the XFM minimal medium, used as a control. Conversely, in grapevine, the bacterial growth increased linearly with the xylem sap concentration. Biofilm production in both citrus and grapevine xylem saps was in line with the recorded bacterial growth. In grapevine xylem sap, biofilm formation linearly followed bacterial growth at each xylem sap concentration: y = 0.3078x - 0.0211 and R² = 0.9089. These results are in line with those reported by Zaini et al. (2009), who found a direct relationship between bacterial growth and the xylem sap concentration in the culture medium using the xylem sap of *Vitis riparia* to cultivate *X. fastidiosa* subsp. *fastidiosa*. Biofilm formation was also directly related to xylem sap concentration in the range 0-90%.

Regardless of the xylem sap concentration, the lowest values of biofilm:growth ratio were recorded in citrus and grapevine (immune species to *Xfp*-ST53). On the contrary, the highest values were observed when the culture media contained 20% xylem saps of both olive cultivars. However, while this ratio sharply decreased with the increase in concentration of the Cellina di Nardò xylem sap, it followed a less dramatic decrease with the increase of the sap concentration of Leccino. This may suggest that, in Leccino (resistant cultivar), the pathogen undergoes rapid cell maturation with the production of biofilm and cell aggregates at the early stage of the infections, hampering the systemic bacterial spread. This is in line with the poor colonization observed in the Leccino plants following their artificial inoculation (Saponari et al., 2017). Conversely, in the susceptible cultivar Cellina di Nardò, the planktonic fraction predominates, resulting in rapid colonization of the host. Therefore, the behavior recorded *in vitro*, besides reflecting the *in vivo* behavior of *Xfp*-ST53 in olives, supports the hypothesis that bacterial virulence is linked to its ability to colonize the host, as shown in grapevine (Hopkins, 1984; Fry, 1990; Ionescu et al., 2013) where xylem colonization of PD-resistant cultivars is limited compared to that occurring in PD-susceptible cultivars. As such, our study showed that the composition of the xylem sap of the resistant cultivar Leccino inhibits the planktonic growth and most likely is part of the multifactorial resistance response recorded in this cultivar.

A set of virulence genes is upregulated by *Xfp*-ST53 when the culture media contain xylem sap, regardless of the plant species from which it originates. This peculiar molecular behavior is demonstrated by the induced expression of cvaC, an antimicrobial peptide that likely facilitates *Xfp*-ST53 invasion of the xylem ecological niche by reducing microbial diversity and algH. The role of this gene is not yet clearly characterized for *X. fastidiosa*, while it is known to be involved in the biosynthesis of alginate, a protecting exopolysaccharide, in *Pseudomonas aeruginosa* and other bacteria. Indeed, algH has been also shown to have a global regulatory function to orchestrate the expression of virulence traits. Additional virulence-associated genes, which were upregulated upon amendment of the media with 80% of olive xylem sap, were PD1560, a nucleotidyltransferase family protein, a not yet characterized virulence protein (Shi et al., 2013), and two well-known virulence proteins, rpfC and pglA. All other genes, particularly those related to biofilm formation, did not vary or were slightly downregulated across the plant species tested. Therefore, the likely emerging scenario is that, at an advanced stage of growth, *Xfp*-ST53 devotes all its effort to virulence, either directed against other bacteria (cvaC) or to systemic spread (pglA).

The NMR-based metabolomic study helped to shed light on the differences in the metabolic composition of the xylem samples extracted from trees belonging to the three species under investigation. The metabolic profile of the sap extracted from the two Apulian olive cultivars reflects those identified for the Picual and Arbequina cultivars (Anguita-Maeso et al., 2021), containing mainly alcohols organic acids, organic acids, carbohydrates, sugar alcohols, and amino acids. Differently from this report a lower amino acids number were found. Among the possible factors causing differences in the metabolic profile is the time of sampling. Indeed saps were extracted in June, when shoot development is almost terminated and mobilization of these compounds is drastically reduced (Sauter, 1981). Mannitol is a major compound of the olive saps of both cultivars,. Its predominance is likely due to the adult age of the plants and the open field conditions of cultivation, as also reported by Anguita-Maeso et al. (2021). Indeed the latter authors found lower amount of this osmolyte in young potted plants, likely subjected to better conditions of irrigation. Some species-specific metabolites have been identified and confirmed through data reported in the literature, such as mannitol and quinic acid for olive (Girelli et al., 2021), tartaric acid for grapevine (Burbidge et al., 2021), and choline for citrus (Servillo et al., 2011). The significant presence of polyols in the xylem of olive and grape could justify the tendency observed in these cases to produce a greater quantity of biofilm as the xylem concentration increases. Biofilm is a matrix composed of cell aggregates, nucleic acids, proteins, and exopolysaccharides (EPS) (Roper et al., 2007). Given its composition, biofilm allows the bacterium to survive in unfavorable conditions, including the presence of antimicrobial compounds (Vestby et al., 2020) or unsuitable osmotic pressure (Padgett-Pagliai et al., 2022). Based on the experimental information collected in this study, the greater abundance of osmolytes in the xylem saps of Leccino and grapevine could trigger the formation of bacterial biofilm to escape osmotic stress.

Once the most significant features characterizing the different species had been identified, a spectroscopic study was undertaken to detect the metabolic variations between the xylem samples obtained from immune and non-immune crops, respectively. In general, xylem sap extracted from immune plants (citrus and grapevine) was more abundant in amino acids, organic acids, and glucose. This evidence is in agreement with the data reported in the literature according to which the main primary metabolites linked to plant defense belong to the classes of carbohydrates, organic acids, amines/amino acids, and lipids (Rojas et al., 2014). A relatively lower concentration of these metabolites was found in the sap extracted from both cultivars of olive trees. However, a slightly higher content of amino acids (alanine and glutamine), organic acids (quinic and malic acids), and carbohydrates (glucose and sucrose) was found in the xylem sap recovered from Cellina di Nardò (*Xfp*-susceptible). The relatively lower content of these metabolites in the *Xfp*-resistant cultivar, Leccino, is in agreement with the results of a study conducted on xylem sap extracted from resistant grape cultivars for which a lower nutrient content was found (Basha et al., 2010).

Mannitol is reported to play an important role in plant response to biotic and abiotic stresses (Stoop et al., 1996). This sugar alcohol is a major osmolyte produced during drought and salt stresses in olive (Gucci et al., 1998; Tsamir-Rimon et al., 2021) that also protects chloroplasts from oxidative stress (Shen et al., 1997). The higher content of mannitol in the resistant Leccino was already reported in metabolome studies of leaf tissues (Scortichini et al., 2021), while an increase of ROS production was observed upon *X. fastidiosa* infections in the trees of Leccino, suggesting a major role in the resistance displayed by this cultivar (Novelli et al., 2019).

In conclusion, through this multidisciplinary study, it was possible to compile some clues on the host-pathogen interaction in OQSD looking at the peculiar features of the xylem sap which represents the niche where *Xylella fastidiosa* subsp. *pauca*, ST53 harbors *in planta*. The combination of omics with *in vitro* studies helped to extrapolate clues on the effect of the metabolic composition of xylem sap on the different behaviors of crops to *Xfp-*ST53, i.e. immune, resistant, and susceptible. A set of primary and secondary metabolites were identified as molecules actively involved in the plant response to this xylem-invading pathogen, likely affecting *Xfp*-ST53 growth rate and biofilm production. Our study showed that bacterial cells were induced to overproduce biofilm rather than multiply in the planktonic phase in the presence of increasing amounts of xylem sap extracted from *Xfp-*immune (*V. vinifera* cv. *Cardinal N.*) and *Xfp-*resistant (*Olea europaea* cv. Leccino) plants. The metabolic study also showed that in these cases the concentration of polyol compounds, such as mannitol, quinic, and tartaric acids was relatively higher than in the *Xfp-*susceptible crop (*Olea europaea* cv. Cellina di Nardò). These data could suggest that one of the strategies adopted by plants capable of surviving in this pathosystem may consist of the overproduction of osmoprotectants and the formation of polymeric structures capable of ensuring the hydration of the vessel. Moreover, the possible exploitations of this knowledge in olive could consist, from the plant side, to the application of osmolytes to mitigate the symptoms, and, from the bacterial side, to induce the biofilm formation to the detriment of the planktonic forms, a strategy already proposed by Chatterjee et al. (2008). Considering the low bacterial growth observed in media amended with xylem sap derived from citrus plants, more studies are needed to disclose the host traits determining immunity to this specific xylem-invading pathogen. The set of genes studied in the transcriptome analysis allowed to better understand how *Xfp* perceives the presence of a given plant species, i.e. differently depending on the level of immunity, susceptibility, or resistance. Further studies are needed to define the plant-pathogen interaction following infection, to better understanding host immunity/resistance mechanisms. Our interdisciplinary study outlines the methodology and paves the way for broader knowledge on the influence of xylem sap features on the host-pathogen interaction in OQDS, confirming the multifaceted nature of this phytopathology.

## Data availability statement

The original contributions presented in the study are included in the article/**Supplementary Material**, further inquiries can be directed to the corresponding author/s.

## Author contributions

AS: Conceptualization, Data curation, Formal analysis, Investigation, Methodology, Writing – original draft, Writing – review & editing. CG: Conceptualization, Formal analysis, Investigation, Methodology, Writing – review & editing. BM: Conceptualization, Data curation, Formal analysis, Funding acquisition, Investigation, Methodology, Writing – original draft, Writing – review & editing. ST: Conceptualization, Formal analysis, Investigation, Methodology, Supervision, Writing – review & editing. AG: Conceptualization, Formal analysis, Investigation, Methodology, Writing – review & editing. GA: Formal analysis, Investigation, Methodology, Writing – review & editing. MS: Conceptualization, Formal analysis, Funding acquisition, Investigation, Methodology, Project administration, Supervision, Writing – original draft, Writing – review & editing. VG: Conceptualization, Formal analysis, Funding acquisition, Methodology, Project administration, Supervision, Writing – original draft, Writing – review & editing. PM: Funding acquisition, Project administration, Supervision, Writing – review & editing. DB: Funding acquisition, Project administration, Supervision, Writing – review & editing. PS: Conceptualization, Formal analysis, Funding acquisition, Methodology, Supervision, Writing – original draft, Writing – review & editing.
