# Supplementary material for: Exploring the xylem-sap to unravel biological features of Xylella fastidiosa subspecies pauca ST53 in immune, resistant and susceptible crop species through metabolomics and in vitro studies
Source: Front Plant Sci. 2024 Jan 19;14:1343876. doi: 10.3389/fpls.2023.1343876 (PMC10834688; doi:10.3389/fpls.2023.1343876)
Supplement: Supplementary file 1 [file DataSheet_1.docx]

Supplementary Material

Exploring the xylem-sap to unravel biological features of *Xylella fastidiosa* subspecies *pauca* ST53 in immune, resistant and susceptible crop species through metabolomics and *in vitro* studies

Antony Surano^1^, Carmine del Grosso^1^, Biagia Musio^2*^, Stefano Todisco^2^, Annalisa Giampetruzzi^1^, Giuseppe Altamura^3^, Maria Saponari^1^, Vito Gallo^2,4^, Piero Mastrorilli^2,4^, Donato Boscia^1^, Pasquale Saldarelli^1*^

*** Correspondence:** Biagia Musio: [biagia.musio@poliba.it](mailto:biagia.musio@poliba.it); Pasquale Saldarelli; [pasquale.saldarelli@ipsp.cnr.it](mailto:pasquale.saldarelli@ipsp.cnr.it)

# Supplementary Figures and Tables

Table S1. Primer sequences used for gene expression analysis on *Xylella fastidiosa* subsp. *pauca* ST53.

| **Primer Name** | **Sequence (5’-3’)** | **Reference** |
| --- | --- | --- |
| acvB For | GACGTCGGCACCAAATGAAT | This study |
| acvB Rev | AGCCTGCGTTACTTCTGGAA |  |
| algH For | GCTGATGCCTCATTGCTGTT | This study |
| algH Rev | TGCCCGGTATAGTCTGTCAA |  |
| cvaC For | ACGGGTTCCACCCCAGAT | This study |
| cvaC Rev | CGGCTTAACGCAGCTATCGT |  |
| fimA For | AGCTCGTACACCGTTCACCAT | (Merfa *et al.*, 2016) |
| fimA Rev | CTGGGCCTGGCTCAAAATAG |  |
| gumB For | TTACCGTGACTGGTGCAGTG | (Merfa *et al*., 2016) |
| gumB Rev | AGACTCGCCAGCGTGTTTAT |  |
| hsf For | GCCGACTGACCCTGATAACT | This study |
| hsf Rev | CGGATGGGATGGTAGGGAAT |  |
| hxfA For | TTCTGACTACGCCTGCCAA | This study |
| hxfA Rev | GATTTAACCCAAAAACCGCC |  |
| hxfB For | ACACCCACAGCTCCCACTAC | (Beaulieu *et al.*, 2013) |
| hxfB Rev | TACCGGCAGCATCTACGTTG |  |
| lipB For | CCTCTCCAACCGCAACTTTC | This study |
| lipB Rev | GGTGTCTGGTACTGGTTCGA |  |
| pcp For | GACACCACCCAAATCAGCTT | This study |
| pcp Rev | GCGGGCAATACGATTCAGAA |  |
| pglA For | GGGTGACGTTGTGGATTGAC | This study |
| pglA Rev | CGCGGAGATCAATGGCATAC |  |
| pilG For | TCGACTCCAGTGATCATGCT | This study |
| pilG Rev | ACTCCTCGCGTGTAAAAGGT |  |
| rpfC For | ACTGCCAATCACCTCCTTCA | This study |
| rpfC Rev | CACAGCGTGTTGTCCTTGAT |  |
| xadA3 For | TGAAACCCGCACAGTGAGTA | This study |
| xadA3 Rev | ATCTTGAGCCACCCGATCAA |  |
| PD1560 For | GCCTTCGTTGCTGTAGAACA | This study |
| PD1560 Rev | CGTTGGGCATCAGTGAAGTT |  |


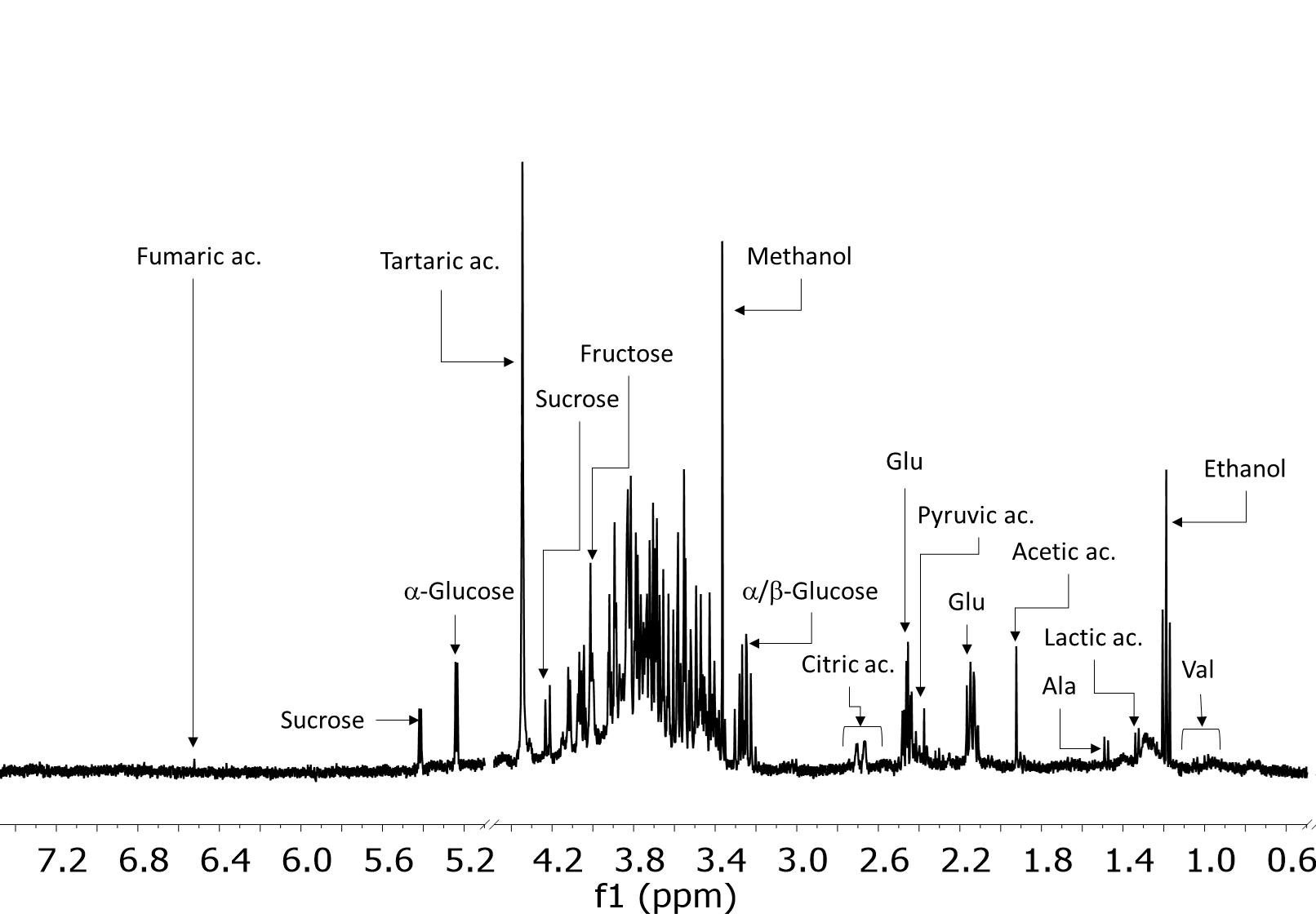


Figure S1. 1D ^1^H NOESY spectrum of xylem sap from *Vitis vinifera* cv. Cardinal N. (Bruker Avance 400 MHz, D_2_O). The assignment of NMR signals has been obtained by comparison with standard compounds. Residual water signal (4.78 ppm) is hidden. Amino acids are indicated as follows: glutamic acid (Glu); Alanine (Ala); Valine (Val).

Table S2. List of metabolites contained in the xylem sap from *Vitis vinifera* cv. Cardinal N. and identified by 1D ^1^H NOESY measurements.

| **Compound** | **d (ppm)** | **Multiplicity** | **J (Hz)** |
| --- | --- | --- | --- |
| *Alcohols* | | | |
| **Ethanol** | 1.19 | t | 6.5 |
|  | 3.66 | q | 6.5 |
| **Methanol** | 3.37 | s |  |
| *Organic acids* | | | |
| **Lactic acid** | 1.34 | d | 6.9 |
|  | 4.16 | q | 6.9 |
| **Citric acid** | 2.67 | d | 15.0 |
|  | 2.71 | d | 15.5 |
| **Acetic acid** | 1.92 | s |  |
| **Fumaric acid** | 6.52 | s |  |
| **Pyruvic acid** | 2.38 | s |  |
| **Tartaric acid** | 4.34 | s |  |
| *Carbohydrates* | | | |
| **Glucose** | 3.24 | dd | 9.1; 7.9 |
|  | 3.42 | m |  |
|  | 3.47 | m |  |
|  | 3.55 | m |  |
|  | 3.74 | m |  |
|  | 3.82 | m |  |
|  | 3.90 | dd | 12.3; 2.1 |
|  | 4.65 | d | 7.9 |
|  | 5.24 | d | 3.7 |
| **Sucrose** | 3.48 | t | 9.2 |
|  | 3.57 | dd | 9.9;3.7 |
|  | 3.67 | s |  |
|  | 3.78 | t | 9 |
|  | 3.83 | m |  |
|  | 3.87 | m |  |
|  | 3.91 | dd | 6.2; 3.5 |
|  | 4.05 | t | 8.5 |
|  | 4.22 | d | 8.7 |
|  | 5.42 | d | 3.8 |
| **Fructose** | 3.57 | m |  |
|  | 3.72 | m |  |
|  | 3.81 | m |  |
|  | 3.91 | dd | 9.9; 3.4 |
|  | 4.02 | m |  |
|  | 4.11 | m |  |
| *Amino Acids* | | | |
| **Alanine** | 1.48 | d | 7.3 |
|  | 3.79 | q | 7.3 |
| **Glutamic acid** | 2.14 | m |  |
|  | 2.46 | td | 7.5; 2.8 |
| **Valine** | 0.99 | d | 7.0 |
|  | 1.04 | d | 7.0 |


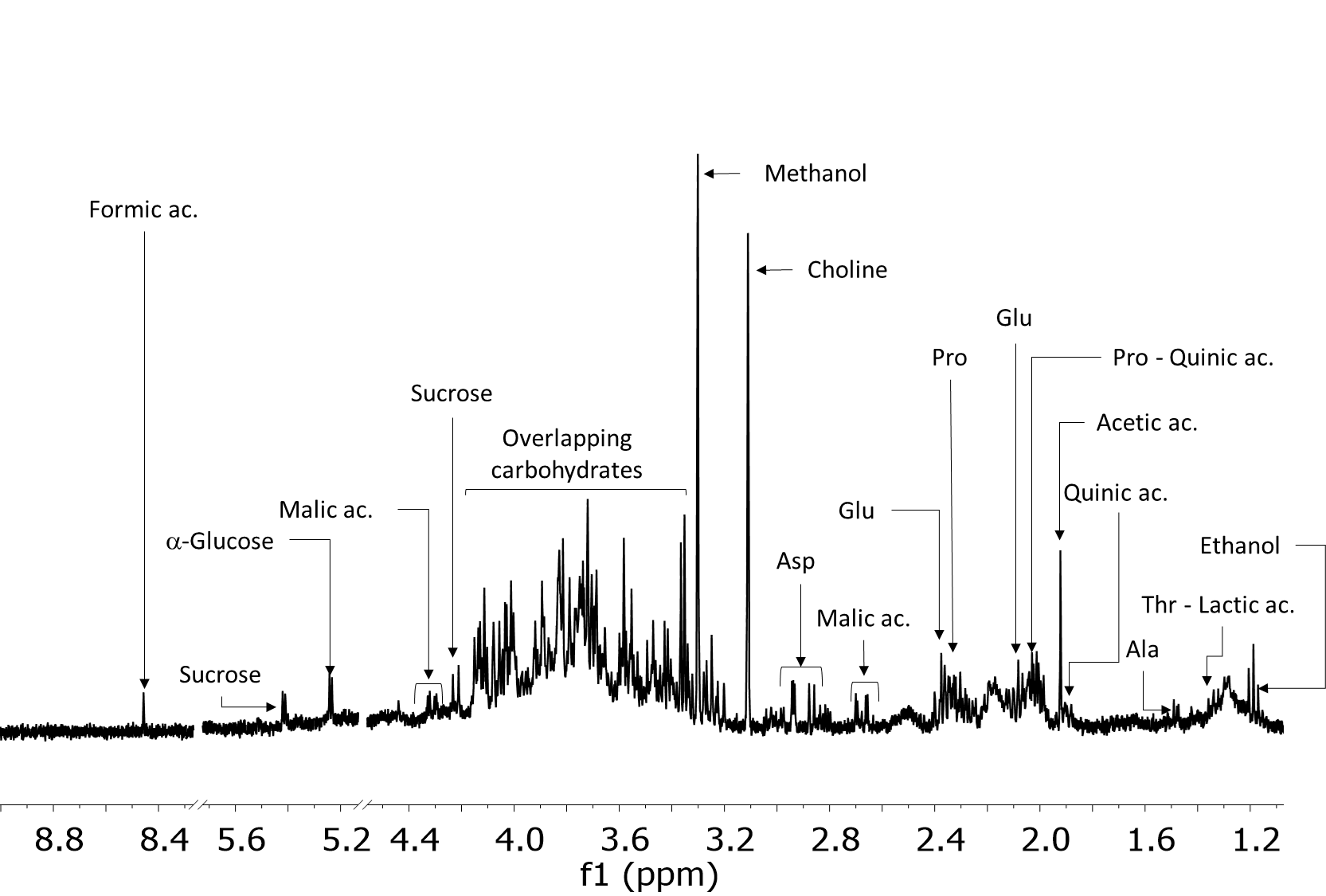


Figure S2. 1D ^1^H NOESY spectrum of xylem sap from *Citrus sinensis* cv. Navelina (Bruker Avance 400 MHz, D_2_O). The assignment of NMR signals has been obtained by comparison with standard compounds. Residual water signal (4.78 ppm) is hidden. Amino acids are indicated as follows: glutamic acid (Glu); alanine (Ala); threonine (Thr); proline (Pro), aspartic acid (Asp).

Table S3. List of metabolites contained in the xylem sap from *Citrus sinensis* cv. Navelina and identified by 1D ^1^H NOESY measurements.

| **Compound** | **d (ppm)** | **Multiplicity** | **J (Hz)** |
| --- | --- | --- | --- |
| *Alcohols* | | | |
| **Ethanol** | 1.19 | t | 6.5 |
|  | 3.66 | q | 6.5 |
| **Methanol** | 3.30 | s |  |
| *Organic acids* | | | |
| **Lactic acid** | 1.33 | d | 6.9 |
|  | 4.13 | q | 6.9 |
| **Malic acid** | 2.67 | dd | 16.3; 4.5 |
|  | 2.81 | dd | 7.5; 4.4 |
|  | 4.31 | m |  |
| **Acetic acid** | 1.92 | s |  |
| **Formic acid** | 8.46 | s |  |
| **Quinic acid** | 1.90 | dd | 13.5; 11.0 |
|  | 2.04 | m |  |
|  | 3.51 | dd | 9.3; 3.3 |
|  | 4.00 | m |  |
|  | 4.16 | q | 3.5 |
| *Carbohydrates* | | | |
| **Glucose** | 3.24 | dd | 9.1; 7.9 |
|  | 3.42 | m |  |
|  | 3.47 | m |  |
|  | 3.55 | m |  |
|  | 3.74 | m |  |
|  | 3.82 | m |  |
|  | 3.90 | dd | 12.3; 2.1 |
|  | 4.65 | d | 7.9 |
|  | 5.24 | d | 3.7 |
| **Sucrose** | 3.48 | t | 9.2 |
|  | 3.57 | dd | 9.9;3.7 |
|  | 3.67 | s |  |
|  | 3.78 | t | 9 |
|  | 3.83 | m |  |
|  | 3.87 | m |  |
|  | 3.91 | dd | 6.2; 3.5 |
|  | 4.05 | t | 8.5 |
|  | 4.22 | d | 8.7 |
|  | 5.42 | d | 3.8 |
| **Fructose** | 3.57 | m |  |
|  | 3.72 | m |  |
|  | 3.81 | m |  |
|  | 3.91 | dd | 9.9; 3.4 |
|  | 4.02 | m |  |
|  | 4.11 | m |  |
| *Amino Acids* | | | |
| **Alanine** | 1.47 | d | 7.3 |
|  | 3.79 | q | 7.3 |
| **Proline** | 2.03 | m |  |
|  | 2.35 | m |  |
|  | 2.39 | m |  |
|  | 4.13 | m |  |
| **Glutamic acid** | 2.06 | m |  |
|  | 2.36 | m |  |
|  | 3.73 | m |  |
| **Threonine** | 1.32 | d | 6.6 |
|  | 3.60 | d | 5.0 |
|  | 4.26 | m |  |
| **Aspartic acid** | 2.85 | dd | 17.7; 7.4 |
|  | 2.93 | dd | 17.7; 4.4 |
|  | 3.99 | dd | 7.4; 4.4 |
| *Quaternary ammonium salts* | | | |
| **Choline** | 3.11 | s |  |
|  | 3.51 | m |  |
|  | 4.06 | m |  |


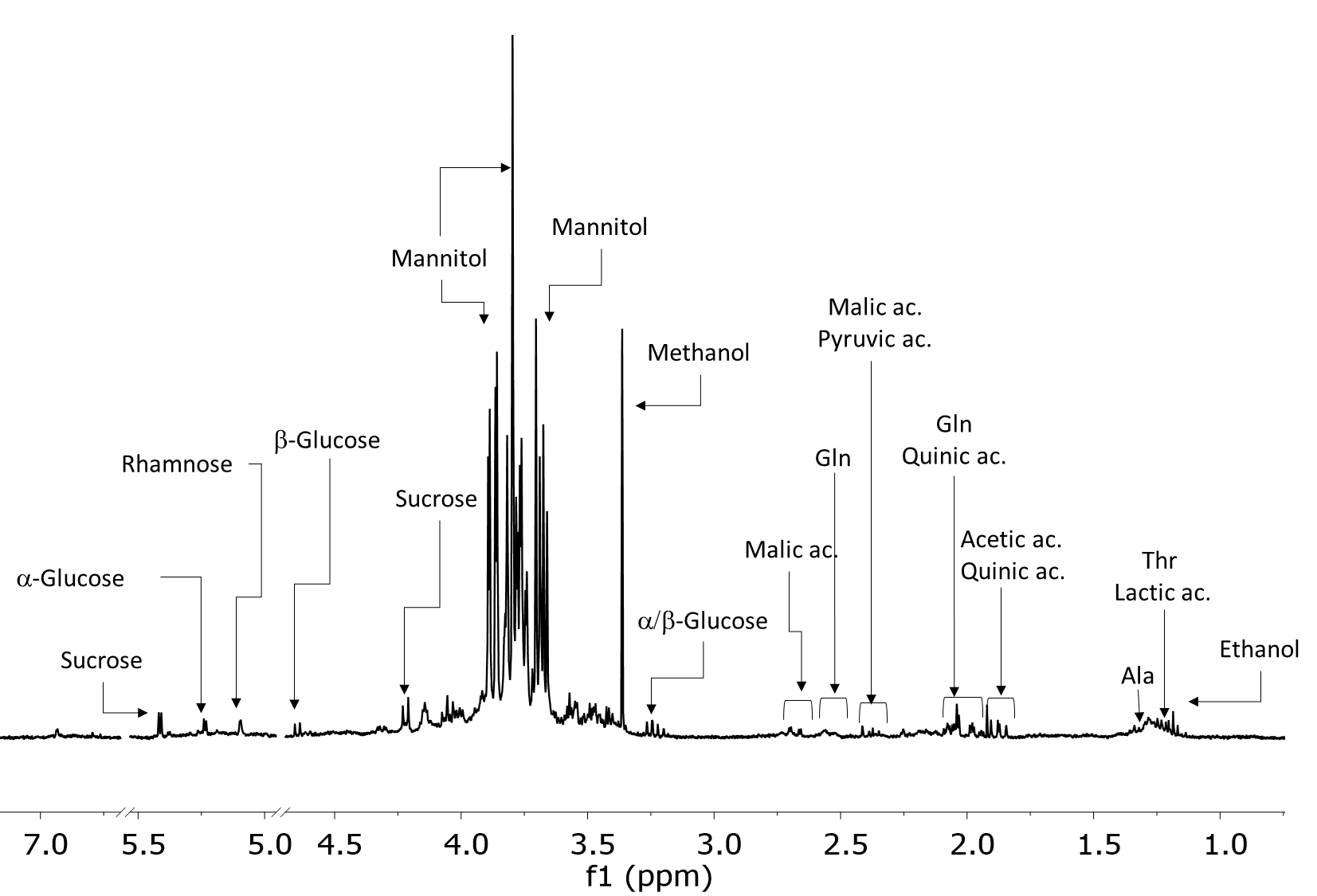


Figure S3. 1D ^1^H NOESY spectrum of xylem sap from *Olea europaea* cv. Leccino (Bruker Avance 400 MHz, D_2_O). The assignment of NMR signals has been obtained by comparison with standard compounds. The residual water signal (4.78 ppm) is hidden. Amino acids are indicated as follows: glutamine (Gln); alanine (Ala); threonine (Thr).

Table S4. List of metabolites contained in the xylem sap from *Olea europaea* cv. Leccino and identified by 1D ^1^H NOESY measurements.

| **Compound** | **d (ppm)** | **Multiplicity** | **J (Hz)** |
| --- | --- | --- | --- |
| *Alcohols* | | | |
| **Ethanol** | 1.19 | t | 6.5 |
|  | 3.66 | q | 6.5 |
| **Methanol** | 3.37 | s |  |
| *Organic acids* | | | |
| **Malic acid** | 2.39 | dd | 16.3; 4.5 |
|  | 2.68 | dd | 7.5; 4.4 |
|  | 4.15 | m |  |
| **Acetic acid** | 1.92 | s |  |
| **Pyruvic acid** | 2.37 | s |  |
| **Quinic acid** | 1.88 | dd | 13.5; 11.0 |
|  | 2.05 | m |  |
|  | 3.56 | dd | 9.3; 3.3 |
|  | 4.04 | m |  |
|  | 4.14 | q | 3.5 |
| *Carbohydrates* | | | |
| **Glucose** | 3.24 | dd | 9.1; 7.9 |
|  | 3.42 | m |  |
|  | 3.47 | m |  |
|  | 3.55 | m |  |
|  | 3.74 | m |  |
|  | 3.82 | m |  |
|  | 3.90 | dd | 12.3; 2.1 |
|  | 4.65 | d | 7.9 |
|  | 5.24 | d | 3.7 |
| **Sucrose** | 3.48 | t | 9.2 |
|  | 3.57 | dd | 9.9;3.7 |
|  | 3.67 | s |  |
|  | 3.78 | t | 9 |
|  | 3.83 | m |  |
|  | 3.87 | m |  |
|  | 3.91 | dd | 6.2; 3.5 |
|  | 4.05 | t | 8.5 |
|  | 4.22 | d | 8.7 |
|  | 5.42 | d | 3.8 |
| **Ramnose** | 1.28 | dd overlapped |  |
|  | 3.40 | m |  |
|  | 3.60 | dd | 9.2; 3.3 |
|  | 3.79 | dd overlapped |  |
|  | 3.86 | m overlapped |  |
|  | 3.92 | m overlapped |  |
|  | 5.09 | d | 1.7 |
| *Sugar alcohols* | | | |
| **Mannitol** | 3.68 | dd | 11.6; 6.2 |
|  | 3.77 | m |  |
|  | 3.81 | d | 8.6 |
|  | 3.88 | dd | 11.6; 2.5 |
| *Amino Acids* | | | |
| **Alanine** | 1.48 | d | 7.3 |
|  | 3.80 | q overlapped | 7.3 |
| **Glutamine** | 2.15 | m |  |
|  | 2.46 | ddd | 7.8; 7.1; 2.2 |
|  | 3.83 | dd overlapped |  |
| **Threonine** | 1.33 | d | 6.6 |
|  | 3.61 | d | 5.0 |
|  | 4.31 | m |  |


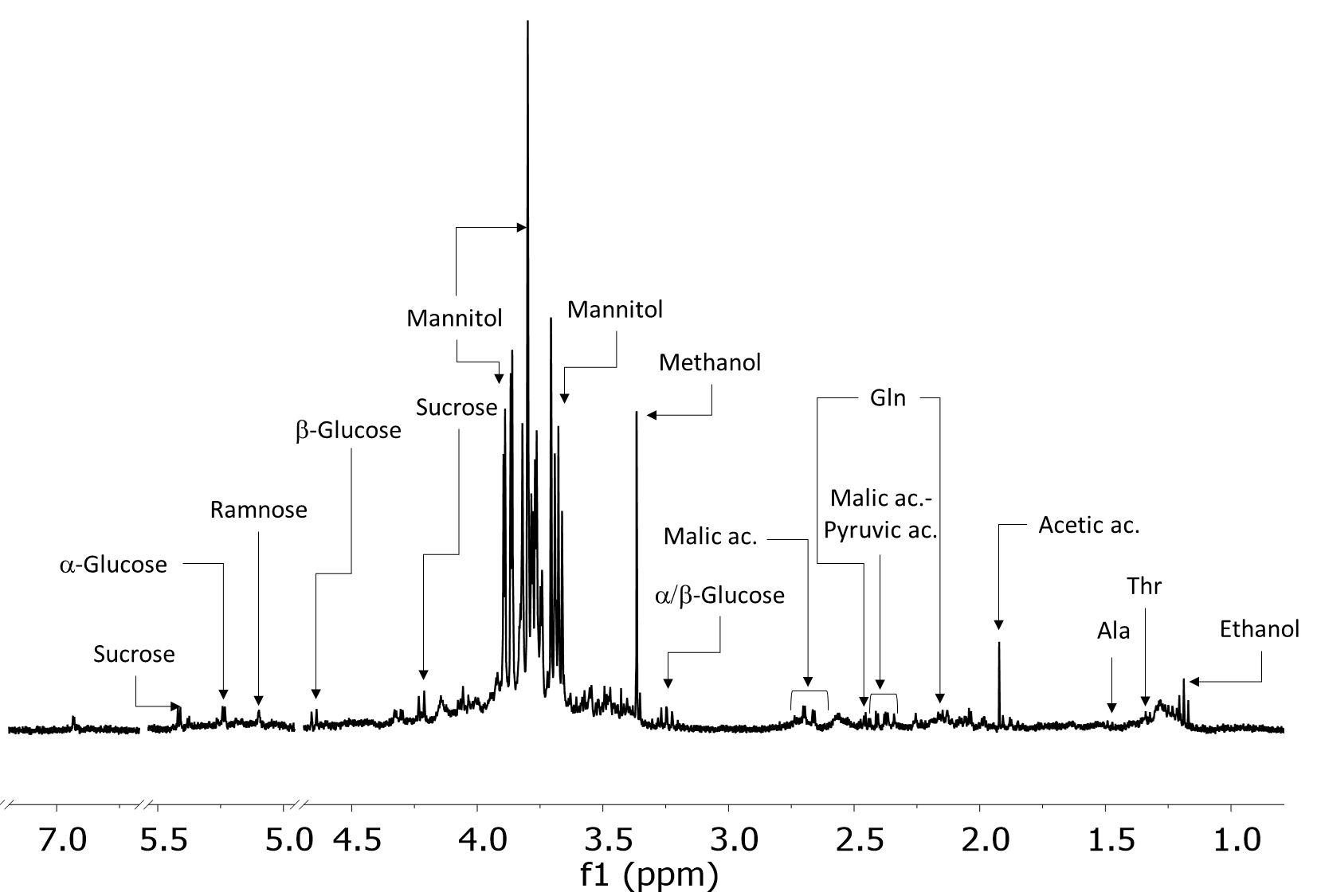


Figure S4. 1D ^1^H NOESY spectrum of xylem sap from *Olea europaea* cv. Cellina di Nardò (Bruker Avance 400 MHz, D_2_O). The assignment of NMR signals has been obtained by comparison with standard compounds. The residual water signal (4.78 ppm) is hidden. Amino acids are indicated as follows: glutamine (Gln); alanine (Ala); threonine (Thr).

Table S5. List of metabolites contained in the xylem sap from *Olea europaea* cv. Cellina di Nardò and identified by 1D ^1^H NOESY measurements.

| **Compound** | **d (ppm)** | **Multiplicity** | **J (Hz)** |
| --- | --- | --- | --- |
| *Alcohols* | | | |
| **Ethanol** | 1.19 | t | 6.5 |
|  | 3.66 | q | 6.5 |
| **Methanol** | 3.37 | s |  |
| *Organic acids* | | | |
| **Malic acid** | 2.39 | dd | 16.3; 4.5 |
|  | 2.68 | dd | 7.5; 4.4 |
|  | 4.15 | m |  |
| **Acetic acid** | 1.92 | s |  |
| **Pyruvic acid** | 2.37 | s |  |
| **Quinic acid** | 1.88 | dd | 13.5; 11.0 |
|  | 2.05 | m |  |
|  | 3.56 | dd | 9.3; 3.3 |
|  | 4.04 | m |  |
|  | 4.14 | q | 3.5 |
| *Carbohydrates* | | | |
| **Glucose** | 3.24 | dd | 9.1; 7.9 |
|  | 3.42 | m |  |
|  | 3.47 | m |  |
|  | 3.55 | m |  |
|  | 3.74 | m |  |
|  | 3.82 | m |  |
|  | 3.90 | dd | 12.3; 2.1 |
|  | 4.65 | d | 7.9 |
|  | 5.24 | d | 3.7 |
| **Sucrose** | 3.48 | t | 9.2 |
|  | 3.57 | dd | 9.9;3.7 |
|  | 3.67 | s |  |
|  | 3.78 | t | 9 |
|  | 3.83 | m |  |
|  | 3.87 | m |  |
|  | 3.91 | dd | 6.2; 3.5 |
|  | 4.05 | t | 8.5 |
|  | 4.22 | d | 8.7 |
|  | 5.42 | d | 3.8 |
| **Ramnose** | 1.28 | dd overlapped |  |
|  | 3.40 | m |  |
|  | 3.60 | dd | 9.2; 3.3 |
|  | 3.79 | dd overlapped |  |
|  | 3.86 | m overlapped |  |
|  | 3.92 | m overlapped |  |
|  | 5.09 | d | 1.7 |
| *Sugar alcohols* | | | |
| **Mannitol** | 3.68 | dd | 11.6; 6.2 |
|  | 3.77 | m |  |
|  | 3.81 | d | 8.6 |
|  | 3.88 | dd | 11.6; 2.5 |
| *Amino Acids* | | | |
| **Alanine** | 1.47 | d | 7.3 |
|  | q | q overlapped | 7.3 |
| **Glutamine** | 2.14 | m |  |
|  | 2.45 | m |  |
|  | 3.73 | t overlapped |  |
| **Threonine** | 1.33 | d | 6.6 |
|  | 3.61 | d | 5.0 |
|  | 4.31 | m |  |

**References**

Merfa, M. V et al. (2016) ‘The MqsRA Toxin-Antitoxin System from Xylella fastidiosa Plays a Key Role in Bacterial Fitness, Pathogenicity, and Persister Cell Formation’, Frontiers in Microbiology, 7:904. doi: 10.3389/fmicb.2016.00904.

Beaulieu, E.D. *et al.* (2013) ‘Characterization of a diffusible signaling factor from Xylella fastidiosa’, *mBio*, 4(1). Available at: https://doi.org/10.1128/mBio.00539-12.
